# An assessment of the impact of the JSY cash transfer program on maternal mortality reduction in Madhya Pradesh, India

**DOI:** 10.3402/gha.v7.24939

**Published:** 2014-12-03

**Authors:** Marie Ng, Archana Misra, Vishal Diwan, Manohar Agnani, Alison Levin-Rector, Ayesha De Costa

**Affiliations:** 1 Division of Global Health, Department of Public Health Sciences, Karolinska Insitutet, Stockholm, Sweden; 2 Institution of Health Metrics and Evaluation, University of Washington, Seattle, WA, USA; 3 Department of Health and Family Welfare, National Rural Health Mission, Government of Madhya Pradesh, Bhopal, India; 4 RD Gardi Medical College, Ujjain, India; 5 Faculty of Education, University of Hong Kong, Pokfulam, Hong Kong

**Keywords:** cash transfer, hospital delivery, maternal health, India

## Abstract

**Background:**

The Indian Janani Suraksha Yojana (JSY) program is a demand-side program in which the state pays women a cash incentive to deliver in an institution, with the aim of reducing maternal mortality. The JSY has had 54 million beneficiaries since inception 7 years ago. Although a number of studies have demonstrated the effect of JSY on coverage, few have examined the direct impact of the program on maternal mortality.

**Objective:**

To study the impact of JSY on maternal mortality in Madhya Pradesh (MP), one of India's largest provinces.

**Design:**

By synthesizing data from various sources, district-level maternal mortality ratios (MMR) from 2005 to 2010 were estimated using a Bayesian spatio-temporal model. Based on these, a mixed effects multilevel regression model was applied to assess the impact of JSY. Specifically, the association between JSY intensity, as reflected by 1) proportion of JSY-supported institutional deliveries, 2) total annual JSY expenditure, and 3) MMR, was examined.

**Results:**

The proportion of all institutional deliveries increased from 23.9% in 2005 to 55.9% in 2010 province-wide. The proportion of JSY-supported institutional deliveries rose from 14% (2005) to 80% (2010). MMR declines in the districts varied from 2 to 35% over this period. Despite the marked increase in JSY-supported delivery, our multilevel models did not detect a significant association between JSY-supported delivery proportions and changes in MMR in the districts. The results from the analysis examining the association between MMR and JSY expenditure are similar.

**Conclusions:**

Our analysis was unable to detect an association between maternal mortality reduction and the JSY in MP. The high proportion of institutional delivery under the program does not seem to have converted to lower mortality outcomes. The lack of significant impact could be related to supply-side constraints. Demand-side programs like JSY will have a limited effect if the supply side is unable to deliver care of adequate quality.

Conditional cash transfer programs initially emerged from Latin America in the late 1990s in response to the social and economic effects of the debt crises of the 1980s (1). These programs provided cash incentives mainly to promote the uptake of preventive maternal/child health services and to improve school attendance among children (2). Despite mixed findings on the effectiveness of these programs in Latin America, similar demand-side incentive programs have become increasingly popular in other low- and middle-income countries around the world in recent years (1). In South Asia, large cash transfer programs (often one-off payments) initiated in Nepal, Bangladesh, and India in the first decade of this century focused mainly on improving access to maternal health services (3). By far the largest of these has been the Indian Janani Suraksha Yojana (JSY) or Safe Motherhood program, which began in 2005.

The JSY, funded fully by the Government of India, is a one-off fixed payment (a single cash transfer) from the state to a woman when she gives birth in a health facility. The criteria for women to qualify as beneficiaries, as well as the size of the cash transfer, vary between states of the Indian Union (4). The JSY, now in its seventh year of implementation, is currently the world's largest cash transfer program with more than 54 million beneficiaries since inception. The objective of the JSY has been to reduce maternal (and neonatal) mortality by promoting in-facility delivery (4). The rationale behind the aim of the program is shown in Fig. 1. The JSY has attracted considerable global interest because of its objective, scale, and scope. Maternal deaths concentrate in disadvantaged groups of women, women who are poor, illiterate, those living in rural, ‘less-developed’ areas, with little or no access to antenatal care, or skilled birth attendance (5).

**Fig. 1:**
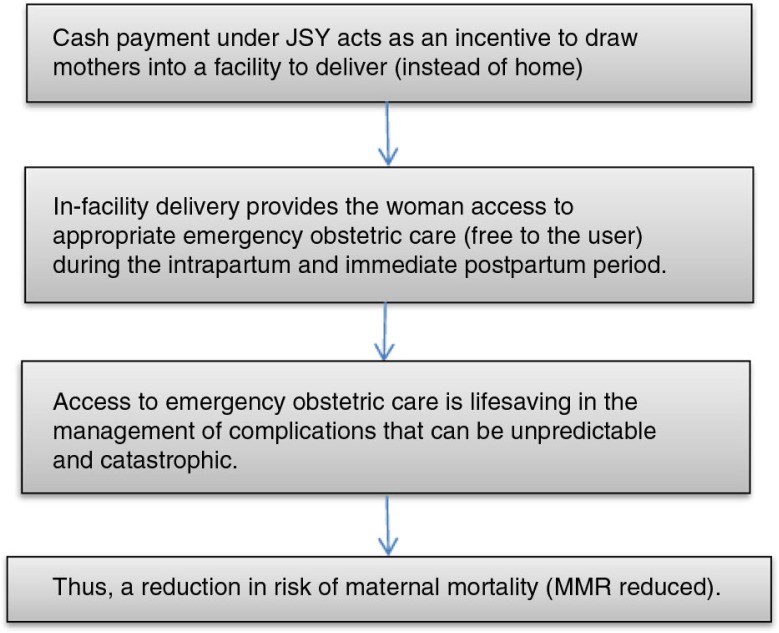
The rationale of the Janani Suraksha Yojana (JSY) program to promote institutional delivery.

National surveys (6–8) have documented a steep increase in institutional delivery proportions since the JSY began, though Lim et al. first reported on the influence of the program on health outcomes (9). Their report based on an analysis of secondary data from the national surveys showed a reduction in perinatal and neonatal mortality rates, though a clear reduction in maternal mortality could not be established. A critique against the study was that it was based on surveys done too soon after the JSY began, and therefore, the definition of JSY beneficiaries did not accurately reflect actual receipt of the JSY benefit but also misclassified mothers receiving cash under other existing schemes as JSY mothers (10). Furthermore, the model used did not take into account heterogeneity in maternal and child care access as well as heterogeneity in program provision across the different districts in India. A second study, also secondary data, showed no effect of the JSY on neonatal mortality (11); it did not look into the effects on maternal mortality. Subsequent studies on the JSY have focused on coverage, processes, and cost reduction (12–14). A recent paper by Randive et al. (15) was unable to detect an association between institutional delivery proportions and MMR using cross-sectional survey data from nine states of India. Seven years after the program began, given the massive investment and high uptake, it is important to study the impact on the main desired outcome, that is, a reduction in maternal mortality (Fig. 1). This is very relevant considering the importance of maternal health in the context of Millennium Development Goals (MDGs) and the fact that many countries in the world are working toward fulfilling MDG 5; India's experience with the cash transfer program is being/will be considered globally for emulation.

In this paper, we 1) describe the uptake of the JSY and the rise in institutional delivery between 2005 and 2010 in the districts of one of India's largest states, 2) estimate the change in district MMR over the same period, and 3) estimate the impact of JSY on the reduction in maternal mortality by examining the association between JSY inputs and the MMR estimates. We reference data from a wide range of sources and synthesize these using modern statistical modeling techniques.

## Methods

### Ethics statement

Ethical approval for the study was obtained from the Institutional Ethical Review Board of the RD Gardi Medical College, Madhya Pradesh (MP).

### Study setting

**Fig. 2:**
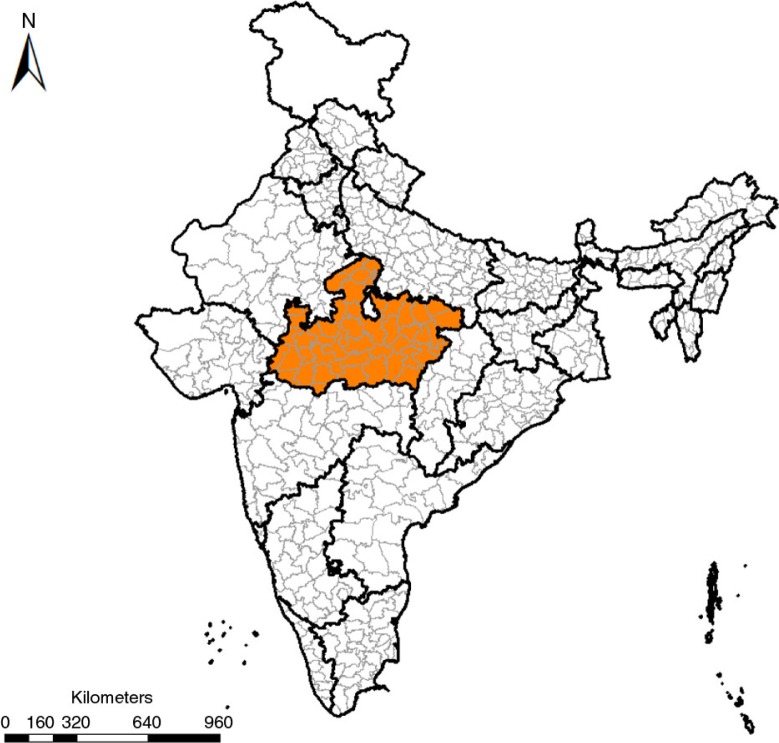
Madhya Pradesh with its 50 districts.

MP is a large central Indian state (Fig. 2); 70% of its 72 million population is rural (16), 37% live below the poverty line (17). Point estimates for MMR reported by two national surveys currently stand between 270 and 310 (18, 19). The state is divided into 50 administrative districts, each with a population of 1–2 million. Each district has its own health administration, which reports program performance within its boundaries. Districts form the units of analysis in this study. In MP, the JSY has functioned almost exclusively through public health sector facilities. All public facilities including the lowest level village sub-centers are JSY facilities; all pregnant women are eligible. Thus far, MP has reported the highest uptake of the JSY program in the country (9).

### Design

We first estimated a continuous time series for MMR in MP from 2005 to 2010 using multiple provincial- and district-level estimates. We then assessed the effect of JSY on MMR by investigating the association between 1) JSY-supported institutional deliveries and MMR, and 2) total annual expenditure on JSY and MMR in the districts of MP.

### Data sources

Maternal mortality data for districts and the state, including the raw number of maternal deaths and maternal mortality ratios (MMRs), were gathered from four major sources: 1) *national government sources*: including SRS reports (1997–2008), Annual Health Survey – 2010, and the National Family Health surveys (1998, 2005); 2) *MP state government sources*: including the vital statistics (2001–2009) and the reports from the provincial Department of Health (2005–2010); 3) *UNICEF report*: specifically, the Maternal and Perinatal Death Inquiry and Response (MAPEDIR) report; and 4) *research studies*: published articles identified via PubMed and Google Scholar Search. Some sources provided direct MMR estimates while others only reported the number of maternal deaths. For the latter, we calculated MMR. The estimated number of live births was derived from the total number of deliveries reported by the Department of Health and then subtracting the number of stillbirths estimated from the stillbirth rates reported in the Annual Vital Statistics. Furthermore, some sources (e.g. NFHS, SRS, AHS) reported maternal deaths at state or district–division level. Considering that these sources are generally regarded to be more reliable than district-level administrative sources, they were applied as an envelope of the district-level estimates. In other words, using state- and division-level numbers as the gold standard, we benchmarked our district-level data such that the sum of maternal deaths among all districts was comparable to the state- and division-level estimates. More details can be found in Supplementary Appendix I Section 1. Socio-demographic covariates including proportion of urban population and the literacy rate among women above 15 years were obtained from the census data (17). In addition, human development indexes were obtained from the three Human Development Reports for the state over the last decade (20–22). Furthermore, fertility rates for each district were obtained from the Office of the Registrar General of India. Program data on the total number of JSY-supported institutional deliveries and JSY-related expenditure from 2005 to 2010 were obtained from the JSY program reports from the districts available through the National Rural Health Mission, Bhopal, MP. Finally, data on the annual proportion of women with three antenatal care visits (ANC3), institutional deliveries, and home deliveries for each district were obtained from the Health Bulletin published by the Department of Health and the District Level Household and Facility Survey (DLHS-3), 2007–2008 (8).

### Models for estimation of MMR

Following the modeling strategy used in several previous studies (23–25), a Bayesian spatio-temporal model (26) was used to estimate complete MMR series from 2005 to 2010 for each district in MP. More specifically, the MMR observed for district *d* from each source *s* in time *t* was assumed to have a negative binomial distribution. The mean of MMR, MMR_*d,t*_, is modeled by

1 $$\log(\text{MM}{\text{R}}_{\text{d},\text{t}})=X_{d[s]}\beta +\alpha_{d[s]}+\zeta_{d[s]}t_{s}+\xi_{d[s],ts}+\delta_{s}$$

The model contains three major components, a covariate component *X*_*s*_ *β*, a time trend component $\alpha_{d[s]}+\zeta_{d[s]}t_{s}+\xi_{d[s],ts},$ and a source-specific effect *δ*_*s*_. The covariate component contains two variables: the total fertility rates and the human development index (a composite index comprising literacy, life expectancy, and income). These two covariates aim to capture the socioeconomic disparities across districts. The district-level MMR trend over time is captured by a linear and a non-linear component. To more accurately estimate the MMR levels and changes, we take advantage of the hierarchical structure in the data and borrow strengths across space and time. More specifically, the changes in MMR in each district are assumed to be nested in those of the regional sub-divisions and the state. In addition, the model captures the variability inherent in different data sets. Estimation of the parameters was performed using the Integrated Nested Laplace Algorithm (27). The posterior distribution of the predicted district-level MMR from 2005 to 2010 was derived. The median MMR was obtained and uncertainty intervals were derived from 2.5th to 97.5th quantiles. Details of the model can be found in Supplementary Appendix I Sections 2 and 3.

Although the above modeling strategy has been widely applied and validated in various contexts (23, 24), limitations in data and model sensitivity are of major concern. Therefore, we conducted a thorough model evaluation to ensure the selected model is indeed the most appropriate for the situation at hand. Five other competing models were investigated. Specifically, we utilized the leave-one-out cross-validation approach and obtained the conditional predictive ordinate (CPO) to examine their predictive validity (28, 29). The results indicate that the model presented above is the best one. Details can be found in Supplementary Appendix I Section 4. In addition, all district-level estimates yielded by the final model were carefully assessed for face validity and the results are presented in Supplementary Appendix I Section 5.

### Impact of JSY on maternal mortality

We assess the impact of JSY on maternal mortality through two sets of multilevel regression analyses.

First, we evaluated the association between JSY-supported institutional deliveries and MMR estimates derived from the Bayesian Model. The regression model is as follows:

2 $$\log(\text{MM}{\text{R}}_{d,t})=\beta_{0}+\beta_{1}\text{Urba}{\text{n}}_{d}+\beta_{2}\text{Li}{\text{t}}_{d}+\beta_{3}\text{ANC}{\text{3}}_{d,t}+\beta_{4}\text{NJS}{\text{Y}}_{d,t}+\beta_{5}\text{JS}{\text{Y}}_{d,t}+\alpha +\varphi_{d}\text{JS}{\text{Y}}_{d,t}$$

The proportion of JSY-supported institutional delivery (JSY_*d,t*_) was estimated by

$$\text{JS}{\text{Y}}_{d,t}=\frac{\text{Total JSY instutitional delivery}}{\text{Total delivery}}$$

The total number of institutional deliveries and home deliveries were obtained from the Health Bulletin reports. However, a comparison between the proportions of institutional deliveries reported by the Health Bulletin and those reported by DLHS-3 in 2007 showed that the former are likely over-reported. Given that survey data may be more reliable than government data, we adjusted the estimated institutional (and home) delivery proportion reported by the Health Bulletin by a correction factor. This factor was based on the ratio of the proportion of institutional delivery reported by Health Bulletin and that reported by DLHS-3. It ranged from 0.34 to 0.97, with a mean of 0.61 (see Supplementary Table 5 for the values of the correction factor applied for each district). Note that the assumption underlying this adjustment was that the bias in Health Bulletin data in 2007 relative to DLHS-3 was consistent across years. As there is only one estimate and no time series in survey estimates over the study period, the adjustment could only be made by benchmarking using 1 year of survey data.

Two demographic covariates, which are related to maternal mortality, are included in the analysis, namely the proportion of urban population (Urban_*d*_) and the literacy rate (Lit_*d*_) among women above 15 years. In addition, the annual proportion of women with *ANC3* was also included to capture health system access at the district level. Furthermore, to capture the secular trend in institutional deliveries, a variable measuring the proportion of non-JSY-supported institutional delivery (NJSY_*d,t*_) was included in the model. NJSY_*d,t*_ was estimated by

$$\text{NJS}{\text{Y}}_{d,t}=\frac{\text{Total instutitional delivery}-\text{JSY instutitional delivery}}{\text{Total delivery}}$$

By incorporating this variable, we aim to differentiate the impact that was attributable to JSY deliveries from that due to the changes in other institutional deliveries.

The random effects *α*_*t*_ and *η*_*d*_ are included in the model to capture the variability in MMR over time across districts which are not accounted for by other factors in the model. Finally, the district-specific slope on JSY-supported institutional deliveries (*φ*_*d*_) was included to capture the heterogeneous impact of JSY across districts.

### Annual JSY expenses and maternal mortality

Second, we evaluated the association between the total annual expenses of JSY and MMR. The reason for considering total annual expenses was to capture the multifaceted nature of JSY. In addition to cash transfers to mothers, JSY consists of other features such as supporting outreach workers (ASHAs) to facilitate birth planning, reimbursing emergency transport costs, compensation to specialists called in when required, and some administrative expenditure. These features (captured here as expenditure) may have an impact on maternal mortality beyond simple institutional delivery. Also, total annual expenses, unlike other program data, may be less prone to misreporting. The regression model is given by

3 $$\log(\text{MM}{\text{R}}_{d,t})=\beta_{0}+\beta_{1}\text{Urba}{\text{n}}_{d}+\beta_{2}\text{Li}{\text{t}}_{d}+\beta_{3}\text{ANC}{\text{3}}_{d,t}+\beta_{4}\text{NJS}{\text{Y}}_{d,t}+\beta_{5}\text{Ex}{\text{p}}_{d,t}+\alpha +\eta_{d}+\varphi_{d}\text{Ex}{\text{p}}_{d,t}$$

The model is similar to the previous one except that the annual total expenditure (Exp_*d,t*_) was considered. JSY annual total expenditure was obtained from the JSY physical report generated by the districts.

In addition to the two models described above, alternative models were considered. They differed in terms of the specification of the random effects component. These models are presented in Supplementary Appendix II Section 3. To take into account uncertainty in the MMR estimates, 1,000 samples of MMR estimates were drawn from the posterior distribution. Models for evaluating the effect of the JSY were fitted to each sample, yielding 1,000 sets of coefficients estimates for each model. The mean coefficient estimates as well as the corresponding uncertainty intervals were derived from the sets of estimates.

All analysis was conducted using R 2.15.0.

## Results

**Fig. 3:**
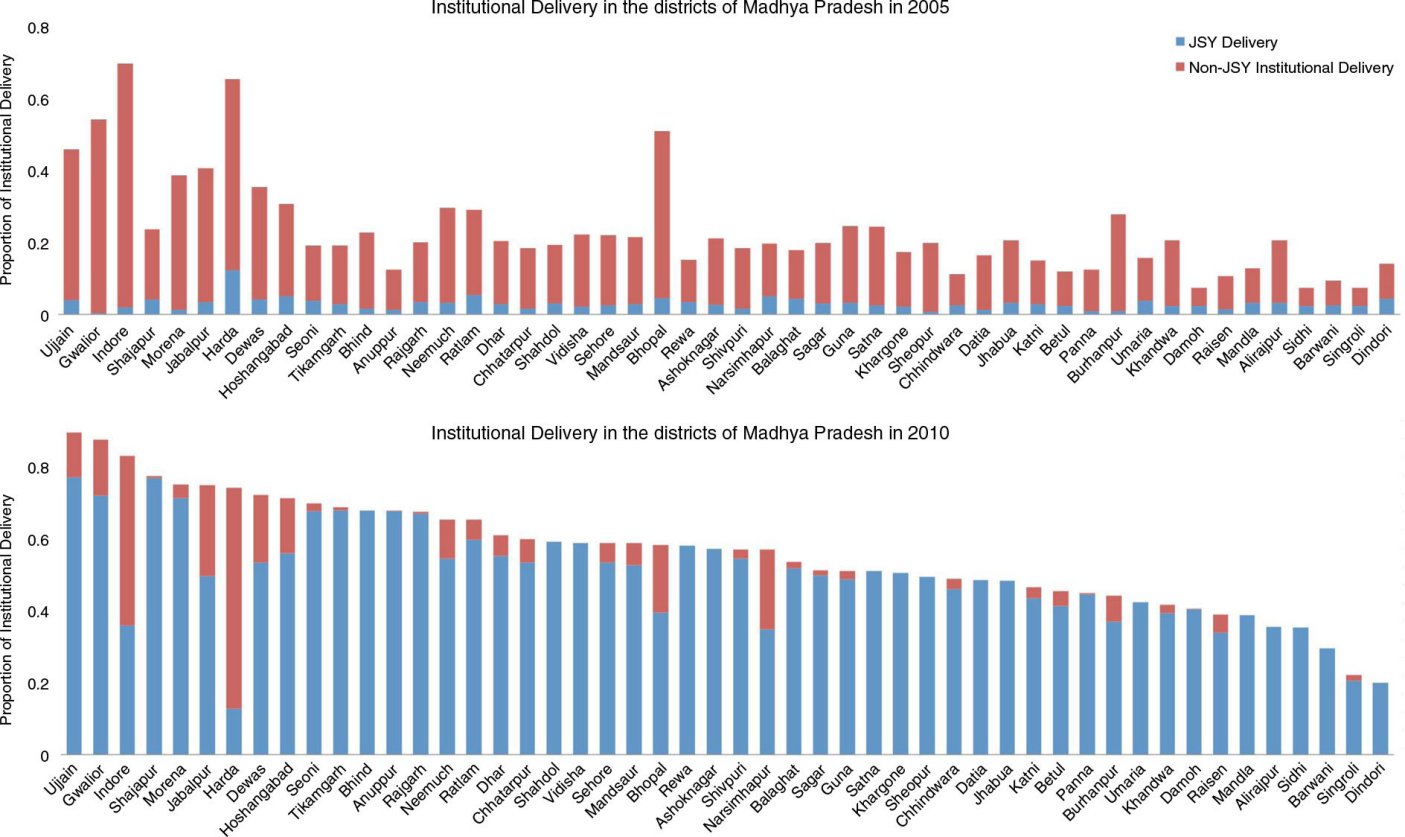
Institutional delivery proportions – Janani Suraksha Yojana (JSY) and non-JSY in the districts of Madhya Pradesh in 2005 and 2010.

**Fig. 4:**
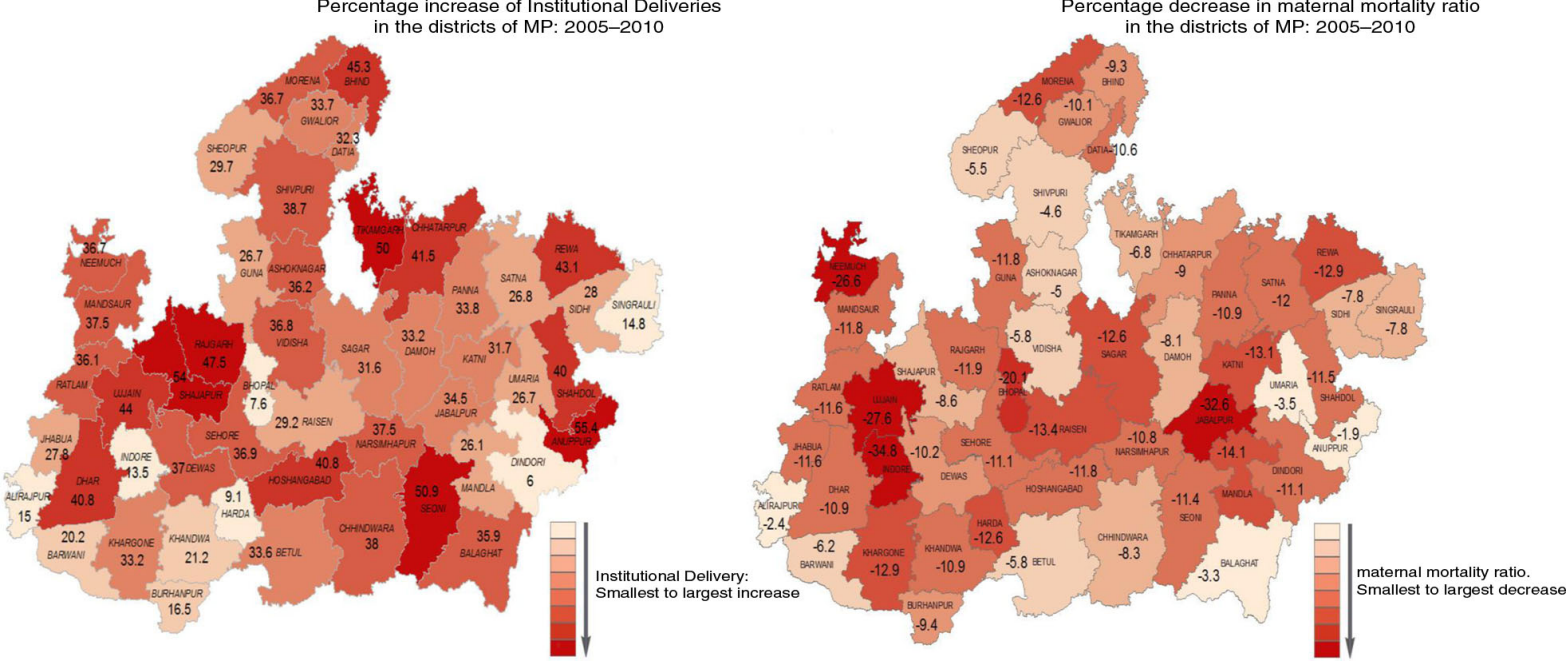
Percentage changes in institutional delivery and maternal mortality ratio (MMR) in the districts of Madhya Pradesh between 2005 and 2010.

We first report the results from our descriptive analysis. The rise in institutional delivery in the districts of MP between 2005 and 2010 is shown in Fig. 3. Changes in maternal mortality and institutional deliveries (both JSY and non-JSY) in the districts of MP between 2005 and 2010 are shown in Fig. 4. For the state as a whole, MMR declined by 12% between 2005 and 2010, from 371 (CI: 241, 511) to 327 (CI: 212, 474) as estimated by our model. Large inter-district variation was observed in the percentage decline ranging from 34.8 to 2%. During the same period, overall institutional deliveries rose from 23.9% in 2005 to 55.9% in 2010 in MP. The increase in institutional deliveries also varied considerably across districts ranging from 7.6 to 54%. The changes in MMR over time and the proportion of institutional delivery in each of the districts are presented in Supplementary Appendix II Section 2.

**Fig. 5:**
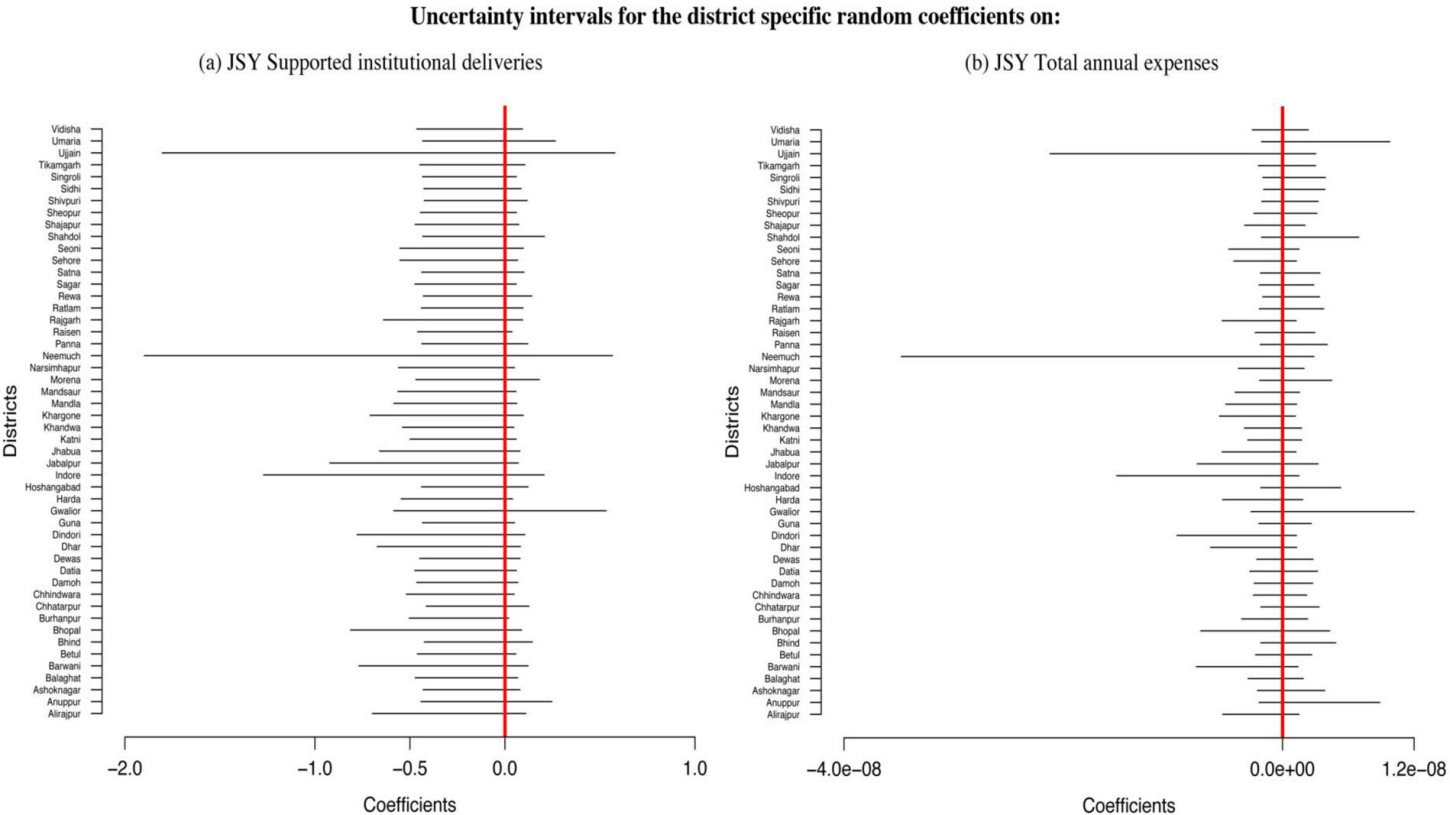
Uncertainty intervals for district-specific random slopes. (a) JSY-supported institutional deliveries; (b) JSY total annual expenses.

Further analysis was carried out using a multilevel regression model to determine whether changes in institutional deliveries supported by JSY were significantly related to changes in MMR. The results are shown in Table 1. The various socio-demographic variables, including proportion of urban population and the literacy rate among women above age 15, were significantly related to MMR decline. Health system access as represented by the proportion of women receiving three antenatal care examinations was not significantly related to MMR. Non-JSY institutional deliveries were negatively related to MMR; however, the association was not significant [−0.219, CI: (−0.537, 0.131)]. Overall, no significant association was found between JSY-supported institutional deliveries and MMR [−0.223, CI: (−0.440, 0.063)]. Examination of the district random slopes (Fig. 5a) indicated that while some variation exists across the districts, none of the districts demonstrated a significant association between MMR and JSY-institutional deliveries.

**Table 1:** Estimated fixed effect coefficients from the multilevel regression model examining the association between maternal mortality ratio (MMR) and Janani Suraksha Yojana (JSY)-supported institutional deliveries

	Estimated coefficients	95% confidence interval	*p*
Intercept	6.752	(6.518, 6.942)	0
Literacy	−0.006	(−0.0106, −0.001)	0.016
Urban	−0.016	(−0.0199, −0.012)	0
≥3 Antenatal care visits	−0.004	(−0.007, 0.001)	0.078
Non-JSY institutional deliveries	−0.219	(−0.537, 0.131)	0.194
JSY-supported institutional deliveries	*−*0.223	(−0.440, 0.063)	0.108

The lack of a significant relationship between JSY-supported institutional deliveries may be due to inadequacy in the metric in capturing the intensity of the program. In order to address this issue, another multilevel regression analysis was carried out using an alternative metric, JSY expenditure. Results are presented in Table 2. As in the previous analysis, proportion of urban population and the literacy rate among women above age 15 in a district were significantly related to MMR decline, while the proportion of women receiving ANC was not. Non-JSY institutional deliveries were negatively associated with MMR but the association was not statistically significant [−0.111, CI: (−0.433, 0.240)]. JSY expenditures were not significantly associated with MMR [0, CI: (−2.488e-06, 1.682e-06)]. Despite considerable variation in the district-specific slopes (Fig. 5b), the effect between JSY expenditure and MMR was not statistically significant in any district.

**Table 2:** Estimated fixed effect coefficients from the multilevel regression model examining the association between maternal mortality ratio (MMR) and Janani Suraksha Yojana (JSY) annual total expenditures

	Estimated coefficients	95% confidence interval	*p*
Intercept	6.734	(6.4926, 6.934)	0
Literacy	−0.007	(−0.011, −0.002)	0.004
Urban	−0.016	(−0.021, −0.012)	0
≥3 Antenatal care visits	−0.004	(−0.007, 0.000)	0.080
Non-JSY institutional deliveries	−0.111	(−0.433, 0.240)	0.486
JSY total annual expenditures (in lakh)	0.000	(−2.488e-06, 1.682e-06)	0.486

## Discussion

Although the JSY has clearly led to an increase in institutional delivery proportions across the state, the ultimate goal of the program is to reduce maternal deaths. However, our analysis was unable to conclusively detect a significant impact of JSY-supported institutional deliveries on a reduction in maternal mortality in the districts of MP. We then conducted a second analysis examining for an association between MMR and JSY annual expenditure, as JSY also provides other support besides the cash transfer, which as discussed earlier could have a potential impact on MMR. As with our findings based on JSY-supported deliveries, the results showed that MMR was not significantly associated with JSY annual expenditure. This again indicates that the impact of JSY on MMR may be limited. Nevertheless, given the various analytical constraints, the lack of significant findings could be a result of a statistical power issue. This is discussed later. However, our results are similar to those reported by Lim et al. (9) and Randive et al. (15).

There are a number of reasons why the program could have had a limited effect on the maternal mortality. While our analysis does not assess these directly, possible explanations are discussed. A major criticism has been the poor quality of care provided to mothers who attend facilities. The public health sector suffers from a serious shortage of key staff required to deliver emergency obstetric care (EmOC), including trained obstetricians, anaesthetists, and nurses (30). These shortages are more acute in rural facilities than in urban ones. There have been reports of facilities unable to deal appropriately with complications, leading to mortality (31). Only a very small proportion of public health facilities have blood storage units or the ability to conduct a caesarean section, both key life-saving functions. Also, as a large proportion of public sector facilities function at less than basic EmOC levels (32), it is likely that a significant proportion of mothers who deliver at these institutions do not receive adequate emergency first-line treatment; some are then referred on to access appropriate care, causing delays and increasing mortality (31). A national evaluation of the program (33) has reported that a large fraction of the institutional deliveries tend to occur disproportionately in only a few facilities, particularly the larger district hospitals. Sharp increases in institutional deliveries at such facilities (13) without a commensurate increase in human resources and infrastructure could lead to compromises in care. Also, the program has experienced challenges to reach the most disadvantaged populations, where maternal deaths are most likely to occur (9). There has been a criticism of the narrow focus on intrapartum care to the neglect of ante- and post-partum care (31, 34). In a recent report of maternal death reviews from MP, 15% of 1,009 mothers who died in the year 2011 did not receive any antenatal care (35), which is an important fact in a state where 59% of pregnant women are anemic (36). Demand-side financing programs with a narrow focus may have limited impact on final goals; thus, if the aim is to reduce maternal mortality and neo-natal mortality, it may be critical to include a range of services like antenatal and post-natal care for mothers, which are critical components in this context (37).

There were large variations between districts, both in the increase in institutional delivery proportions and in MMR reduction, after JSY began. The relatively urban districts with better physical infrastructure (Bhopal, Indore, Ujjain) experienced sharper falls in MMR, though they had relatively moderate increases in institutional delivery. The rural districts that saw a steep rise in institutional delivery proportions experienced only small declines in MMR. It is possible that the comparatively better physical infrastructure in the urban districts mean fewer physical access barriers, which allow easier and earlier access to relatively better functioning facilities (than in rural districts). A continued focus on the development of physical infrastructure in the rural areas of the state as well as a focus on the quality of care in public facilities is likely to contribute to lower MMR.

### Implications for the context and beyond

MP is one of India's nine ‘high-focus’ states, that is, states that require and receive additional resources to improve poor health indicators. These nine states account for three quarters of India's maternal deaths and 12% of all global maternal deaths. An assessment of the impact of JSY in MP is therefore not only of national interest but also of global interest, as other countries look for innovative strategies to progress toward MDG 5.

Though not often stated explicitly, the main argument cited in favor of demand-side financing interventions, including cash transfers, is that beneficiaries face financial barriers that prevent them from using a particular service or intervention (38). Given the sharp rise in institutional delivery uptake, it is likely that the cash transfer did reduce financial access barriers; although, there has been a suggestion that the JSY cash transfer not be viewed as a cash transfer to reduce financial barriers but rather as an incentive to promote a desired behavior (39). However, the implicit assumption that simply raising institutional delivery proportions will translate into a reduction in maternal mortality merits reconsideration. An important qualification is that institutional delivery will reduce maternal deaths if competent skilled birth attendance is provided at these facilities, but is unlikely to otherwise. Skilled birth attendance here is defined as the presence of a skilled attendant (as opposed to a trained attendant, as a trained/qualified attendant, i.e., not necessarily competent or skilled) and an enabling environment which includes adequate supplies, equipment, and infrastructure as well as efficient and effective systems of communication and referral (40). If institutions under the JSY are unable to provide skilled birth attendance, it is unlikely that the program will impact maternal mortality. Though there have been efforts under the National Rural Health Mission to strengthen institutional capacity, more structural changes in the health system (to improve accountability, manage human resources) and in the education of health cadres is called for, to ensure that these institutions are truly capable of delivering life-saving skilled birth attendance.

Demand-side programs have been implemented in developed countries embedded in contexts where populations are well registered, bureaucracies function, and accountability mechanisms are in place. Managing such schemes is more challenging in contexts where these are lacking. These schemes are also not palliatives for serious structural concerns that remain to be addressed. Demand-side programs should not be seen as a ‘quick-fix’ substitute for supply-side interventions. The lack of fair and functioning systems that undermine supply-side interventions will also affect demand-side ones (41). The introduction of a demand-side program like the JSY therefore involves an assessment of the state of the existing services, and potential supply-side investments needed to raise standards prior to inflating demand. This should focus on the accessibility of services, the availability of services (staffing, opening hours, etc.), having adequate infrastructure (equipment, buildings, drugs, etc.), appropriate processes (infection prevention etc.), and management (staff workload, supportive supervision etc.) (42).

There has been some discussion on the role of demand-side programs in stimulating accountability in service delivery (41). Mechanisms such as making the income of health service providers depend more on demand from poor clients, making providers accountable to local bodies for their performance, and fostering the involvement of poor people in the monitoring and provision of services have been suggested (41). Under the National Rural Health Mission, district-level vigilance and monitoring committees have been set up (43). These comprise elected representatives and government officials who are mandated with monitoring the implementation of the JSY. The extent of functioning of these committees and corrective actions taken are not reported, but in the light of evidence (31) their influence seems limited. Quality improvement measures like maternal death reviews have been initiated to improve accountability. The National Rural Health Mission itself has put in place a number of measures to improve quality, equity, and accountability in its programs. However, there have been no objective assessments of the improvements in accountability within the JSY (or any other program) brought about by these measures.

### Methodological discussion

Establishing the impact of JSY on maternal mortality is challenging for several reasons. First, the evaluation is retrospective based on secondary data. Establishing a causal link between the program and maternal mortality is therefore difficult. Results from our models based on observational data therefore need to be interpreted with care. The estimates generated were noisy with, large standard errors which could account for the lack of effect seen in the districts. Second, there is a lack of data on the time trend of maternal mortality at the district level. Although some data are available from government sources, as previously mentioned, MMR data were scanty. We gathered data from multiple sources, each of which suffers from a different bias. Through the use of a Bayesian Model, we attempted to take into account the uncertainty across different sources to generate complete district-level MMR time series. The major strength of the model lies in its ability to take advantage of the geographical and temporal associations within data. However, some of the biases are likely to be present in the estimates. Moreover, the variability in data led to wide uncertainty intervals. This could have contributed to low statistical power and non-detection of an effect on MMR in the models. Third, the quality of program data was uncertain. Specifically, there appeared to be over-reporting of institutional deliveries and JSY beneficiaries in the Health Bulletin and JSY reports. To correct for the systematic bias, we derived an adjustment factor with reference to DLHS-3 reported number of institutional deliveries and JSY coverage. This adjustment factor assumed that the extent of bias differed by district but was constant over time for each district. This assumption, though not ideal, provides a less biased approximation of the true coverage of institutional delivery.

## Conclusions

To sum up, our study confirms previous reports that the proportion of institutional delivery has climbed steeply since the inception of JSY. However, our analysis was unable to conclusively detect an effect of the JSY on maternal mortality reduction. Recent reports have raised questions related to the quality of obstetric care in the program, which could influence mortality outcomes. Demand-side programs are unlikely to be successful in the absence of a well-functioning supply side capable of delivering adequate EmOC. Establishing such demand-side programs in the absence of a well-prepared supply side will prevent program uptake from being converted into lower mortality outcomes. Governments seeking to deploy demand-side financing mechanisms to achieve better maternal health outcomes should approach this circumspectly and, certainly, ensure a well-functioning supply side before initiating a demand-side program.

## Supplementary Material

An assessment of the impact of the JSY cash transfer program on maternal mortality reduction in Madhya Pradesh, India
