# Supplementary material for: An assessment of the impact of the JSY cash transfer program on maternal mortality reduction in Madhya Pradesh, India
Source: Glob Health Action. 2014 Dec 3;7:10.3402/gha.v7.24939. doi: 10.3402/gha.v7.24939 (PMC4256523; doi:10.3402/gha.v7.24939)
Supplement: An assessment of the impact of the JSY cash transfer program on maternal mortality reduction in Madhya Pradesh, India [file GHA-7-24939-s001.pdf]

Webappendix I: Estimation of District-Level Maternal Mortality

1. Data

Maternal mortality data including the raw number of maternal deaths and maternal mortality ratios (MMR) were gathered from four major sources, namely national government sources, state government sources, UNICEF report, and research studies. National government sources include data from the Sample Registration System (SRS), Annual Health Survey (AHS), and National Family Health Survey (NFHS). State government sources include data from Annual Vital Statistics (AVS) and Health Bulletin (HB). UNICEF report includes Maternal and Perinatal Death Inquiry and Response (MAPEDIR) Report. Two research studies were identified via PubMed and Google Scholar Search, namely Ranjan 2004 and De Costa et al. 2009. Webtable 1 provides a summary of all the data sources used in this study, the types, level and years data were extracted.

MMR estimates are provided in SRS, AHS, NFHS, and Ranjan's study. In AVS and Health Bulletin, only the raw numbers of maternal mortality were reported. We calculated the MMR using the number of maternal deaths reported divided by an estimated number of live births. The estimated number of live births was derived from the total number of births reported in the Health Bulletin minus the number of still births estimated from the still birth rates reported by the vital registration.

The sources shown in the table differ in terms of their reliablity, with sources such as NFHS, SRS, AHS generally perceived as being more reliable. However, NFHS, SRS and AHS report maternal mortality at state or district-division level. To capitalize on all the available data sources, we utilized these state- and division-level maternal mortality estimates as an "envelope" of the district-level estimates. Specifically, using the state- and division- level number as the gold-standard, we adjusted the district-level data such that the sum maternal deaths of all districts would be comparable to those of the state- and division- level estimates.

Webtable 1: List of data sources for maternal mortality.

Source categories	Data sets	Types of data	Years	Level
National sources	Sample Registration System (SRS)	MMR	1997, 2000, 2002, 2005, 2008	State
	National Family Health Survey (NFHS)	MMR	1998, 2005	State
	Annual Health Survey (AHS)	MMR	2010	District division
State sources	Annual Vital Statistics (AVS)	Raw deaths	2001-2009	District
	Health Bulletin (HB)	Raw deaths	2005-2010	District
UNICEF reports	UNICEF MAPEDIR Report [1]	Raw deaths	2006-2007	District
Research studies	Ranjan, 2004 [2]	MMR	1997	District
	De Costa et al., 2009 [3]	MMR	2002-2004	District

2. Statistical Model

Given the considerable data sparsity and potential bias in the data sources, a statistical model was used to synthesize the data and to generate a complete MMR time series. In particular, the maternal mortality ratio observed for district *d* in each study *s* (MMR_s) is assumed to have a negative binomial distribution:

$$MMR_s \sim NB(r, p),$$

The mean of MMR, $\mu = r\frac{1-p}{p}$, is modeled by:

$$\log(\mu) = X_s\beta + \alpha_{d[s]} + \zeta_{d[s]}t_s + \xi_{d[s],t_s} + \delta_s \qquad (1)$$

Negative binomial distribution is chosen because of considerable heterogeneity in MMR across districts. Negative binomial is able to capture the overdispersion in the data.

As shown in Equation (1) above, the model contains four major components: a covariate component $X_s\beta$, a time trend component $\alpha_{d[s]} + \zeta_{d[s]}t_s + \xi_{d[s],t_s}$ and a study-specific effect δ_s. The covariate component includes two covariates, namely total fertility rates ($TFR_{d,t}$) and human development index ($HDI_{d,t}$). $TFR_{d,t}$ was obtained from the AVS. It was included in the model as previous studies have indicated TFR to be a reliable predictor for maternal mortality. HDI was obtained from the UN Human Development Report 1998, 2005, and 2007. It is used to capture the varying socioeconomic situation across the districts. HDI is used as a covariate as opposed to other indicators such as percent urbanization or GDP because HDI provide more differentiability across districts. Moreover, within the period of 1997 to 2010, there were at least three distinct estimates for HDI, whereas for other indicators, only one time point was available. Hence, HDI enables us to better capture the socioeconomic changes overtime.

The time trend component consists of two parts, one capturing the overall linear trend, one capturing the nonlinear changes. The linear trend is captured by

$$\alpha_{d[s]} + \zeta_{d[s]}t_s,$$

where α and $\zeta$are the district-specific intercept and slope. Specifically,

$$\alpha_d = \alpha_d^D + \alpha_{r[d]}^R + \alpha^M$$

$$\zeta_d = \zeta_d^D + \zeta^M$$

Three levels of variability are captured: namely state level (*M*), cluster level (*R*), and district level (*D*). The cluster in which a district belongs is based on their level of economic development reflected by the percent of villages with electricity. The district-level percent of villages with electricity was obtained from DLHS-2. According to the data, we ranked and divided the districts into eight clusters. A normal prior is assigned to each component. The non-linear changes over time are captured by $\xi_{d[s],t_s}$, with a random walk prior assigned.

Finally, to capture the variability in the data sets, the random effect (δ_s) is included. In particular, we group the data sets into four source categories: national sources, state sources, UN reports, and research studies. Depending on the category a data set belongs to, the variance of δ_s is defined as

$$Var(\delta_s) = \begin{cases} \nu_n^2 & \text{for Naional source} \\ \nu_s^2 & \text{for State source} \\ \nu_u^2 & \text{for UN reports} \\ \nu_c^2 & \text{for Research studies} \end{cases}$$

where $\nu_s^2 > \nu_c^2 > \nu_u^2 > \nu_n^2$.

3. Estimation of MMR

An outcome of interest here is the district-level MMR from 2005 to 2010. For district *d* in year *t*, we calculate:

$$\hat{\eta}_{d,t} = X_s \hat{\beta} + \alpha_{d[s]} + \zeta_{d[s]} t_s + \xi_{d[s],t_s} + \delta_s \quad (2)$$

$$\widehat{MMR}_{d,t} = \exp(\hat{\eta}_{d,t})$$

where $\widehat{\beta_J}$'s are the estimated fixed effect coefficients and α_d, ζ_d, ξ_{d,t_s} and δ_s are the estimated random effect coefficients. Uncertainty intervals are derived from the 2.5th and 97.5th quantiles of the posterior marginal distribution of the predicted values.

4. Model Validation

4.1 Alternative models

In addition to the model presented above, we also consider six alternative models. Similar to the model presented earlier, alternative models 1 to 6 (AM1-6) all assume that MMR follows a negative binomial distribution. However, they differ in terms of the specification of the covariate component and specification of priors.

AM1 and the original model differ in terms of the specification of prior for the non-linear time trend component. In AM1, instead of a Gaussian autoregressive prior, an order 1 random walk prior was assigned.

In AM2 and 3, the division effect in the time trend component is omitted. More specifically,

$$\alpha_d = \alpha_d^D + \alpha^M$$

$$\zeta_d = \zeta_d^D + \zeta^M$$

$$\xi_{d,t} = \xi_{d,t}^D + \xi_t^M.$$

Different priors were assigned for the non-linear component. For AM2, a Gaussian autoregressive prior was assigned, whereas for AM3, a random walk prior was assigned.

In AM4, $\log(\mu)$ is modeled by

$$\log(\mu) = X_s\beta + \alpha_{d[s]} + \zeta_{d[s]} t_s + \xi_{d[s],t_s} + \delta_s + \phi_{d[s]} \quad (3)$$

Similar to AM2 and 3, the division effect in the time trend component is omitted. However, unlike the previous models, an additional component $\phi_{d[s]}$ is included to capture the spatial relationship across district. $\phi_{d'}$ is assigned a besag prior

$$\phi_{d'} | \phi_l \sim N\left(\frac{1}{n_d} \sum_{d \sim l} \phi_l, \frac{1}{n_d \tau}\right) \quad (4)$$

where n_d refers to the number of neighbours connected with district d. $d \sim l$ refers to neighbouring districts. τ is the precision parameter.

In AM5, $\log(\mu)$ is modeled by

$$\log(\mu) == X_s\beta + \alpha_{d[s]} + \xi_{d[s],t_s} + \delta_s + \phi_{d[s]} \tag{5}$$

where the overall time trend is captured solely by the random effect $\xi_{d[s],t_s}$, which is assigned a random walk of order 1 prior.

For the last alternative model (AM6), $\log(\mu)$ is modeled the same way as the original model Model (3) except that the distribution of MMR is assumed to follow a Poisson distribution rather than a negative binomial.

4.2 Logarithmic scores based on conditional predictive ordinate

To examine the validity of the models, we utilized the leave-one-out cross-validation approach and obtained the conditional predictive ordinate (CPO). There are many alternative methods for evaluating the model validity; however, CPO was adopted here as it is able to take into account the complexity of the model at the same time, and it is not as conservative as posterior predictive check. [1] We compare the fit of the models using the sum of log CPO scores (log-scores). Webtable 2 presents the log scores of the original and alternative models. As indicated in the table, the model based on the negative binomial distribution is superior to the model based on the Poisson distribution, as the log-scores of the original model as well as AM1-5 are markedly smaller than that of AM6. Relative to AM 1-5, the original model offers a slightly better fit.

Webtable 2 Log-scores comparing the alternative models with the original model.

	Original	AM2	AM3	AM4	AM5	AM6
Log scores	-7757	-7897	-7895	-7882	-7928	-25664

5. District level MMR from 2005 to 2010

This section presents the MMR time trends, with CI from 2005 to 2010, for the 50 districts in Madhya Pradesh.

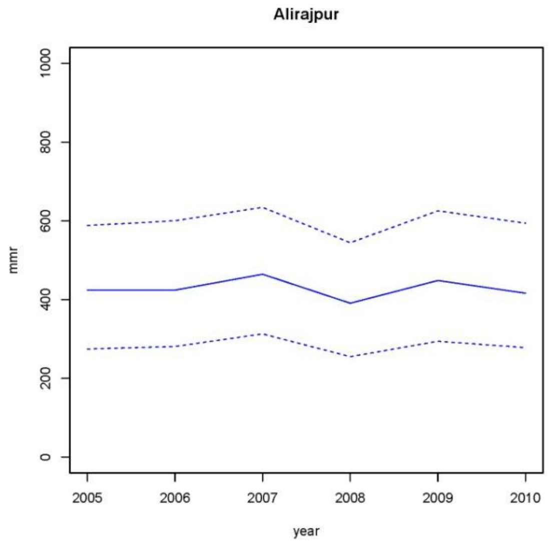

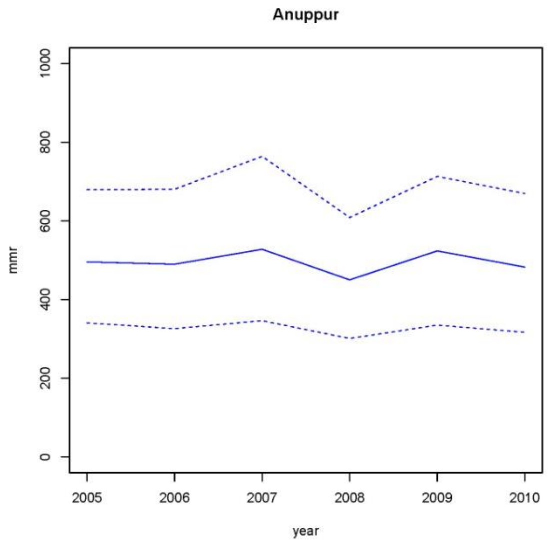

Ashoknagar

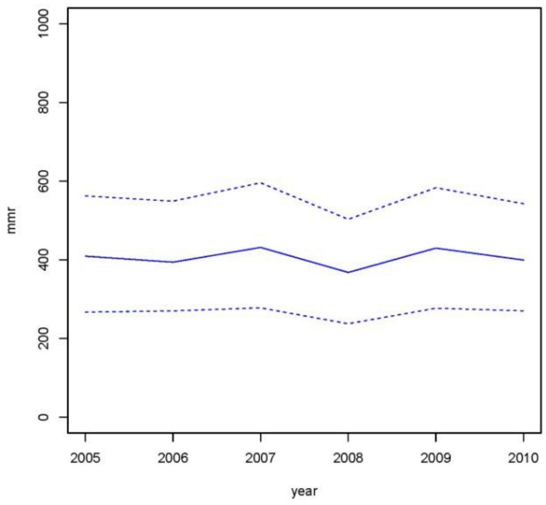

Balaghat

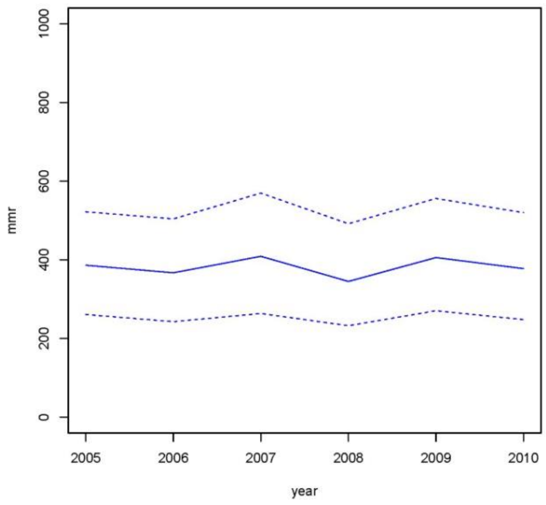

Barwani

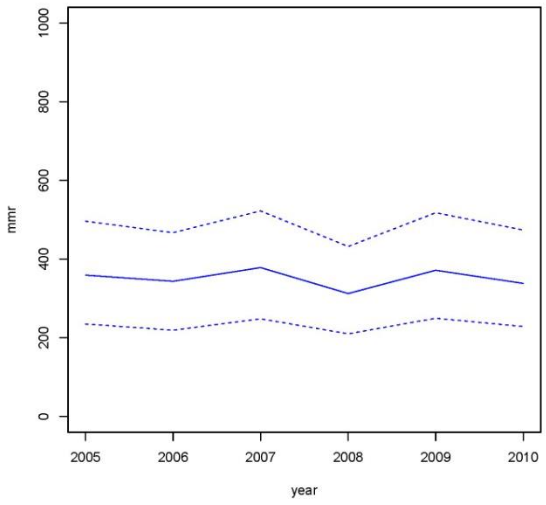

Betul

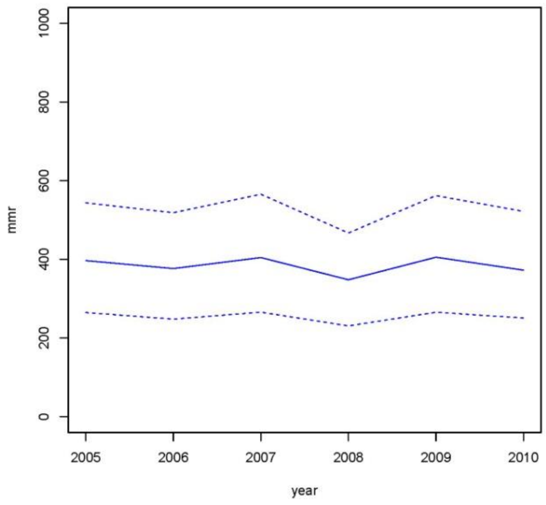

Bhind

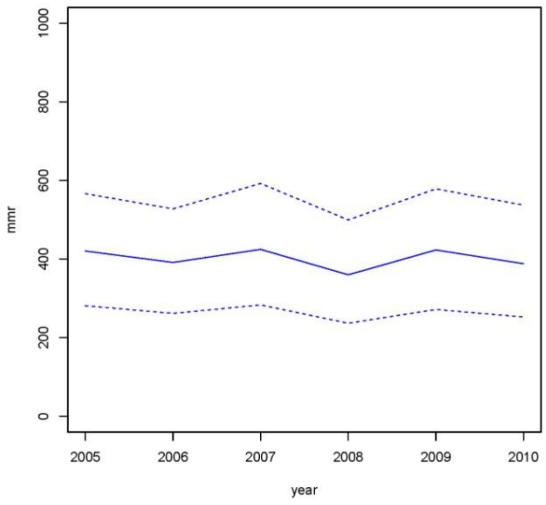

Bhopal

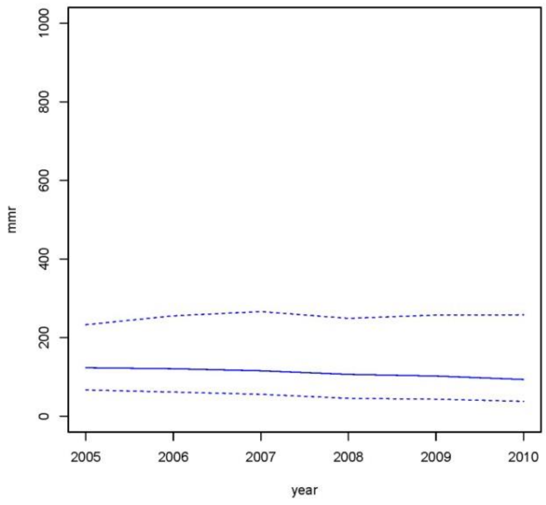

Burhanpur

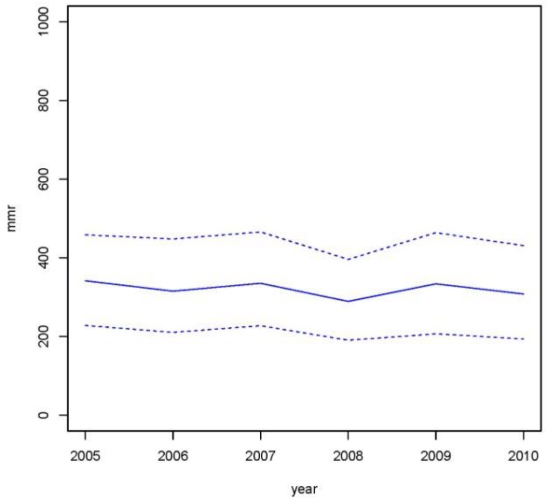

Chhatarpur

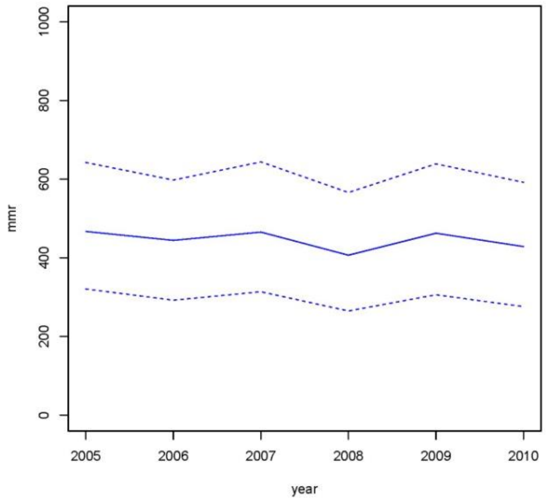

Chhindwara

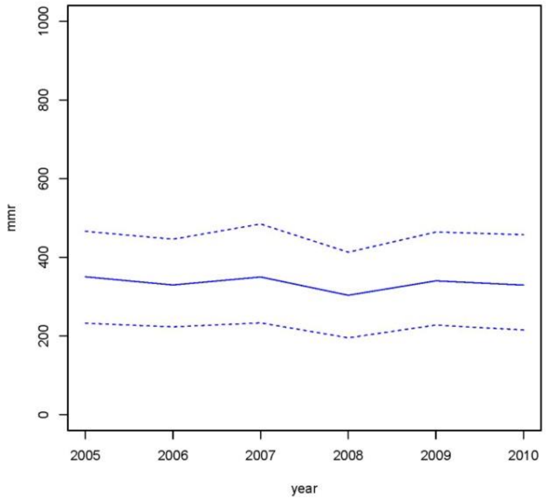

Damoh

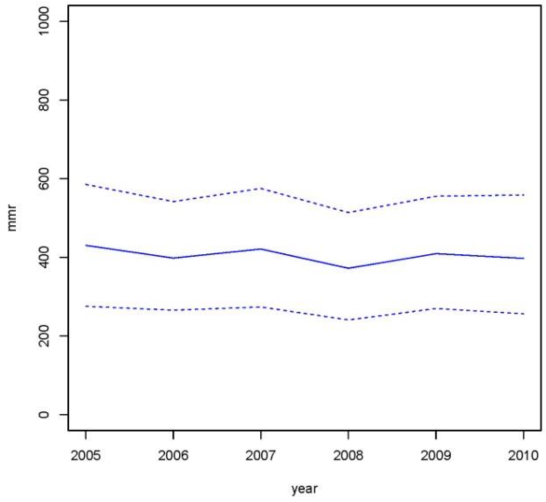

Datia

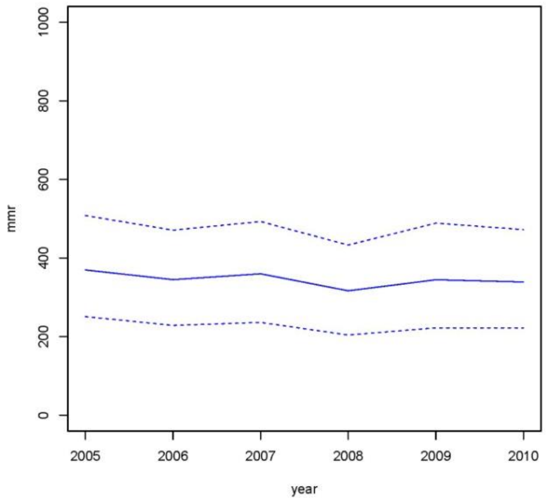

Dewas

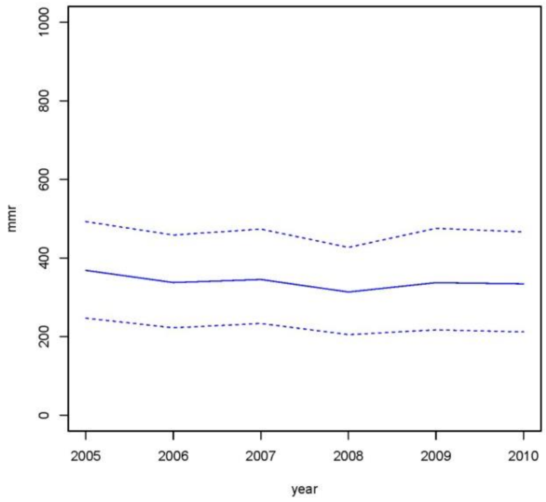

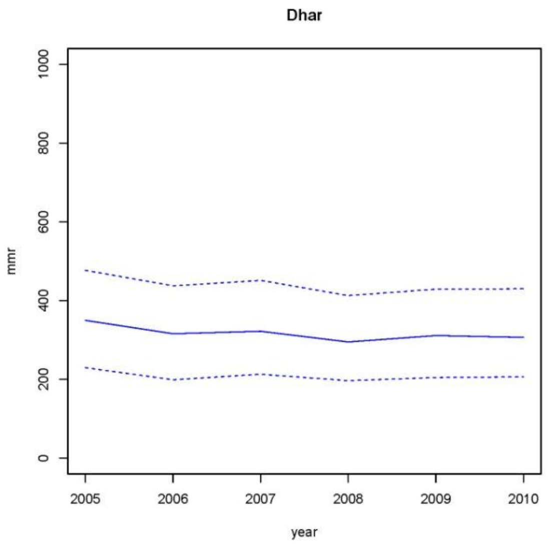
Dhar
mmr
year

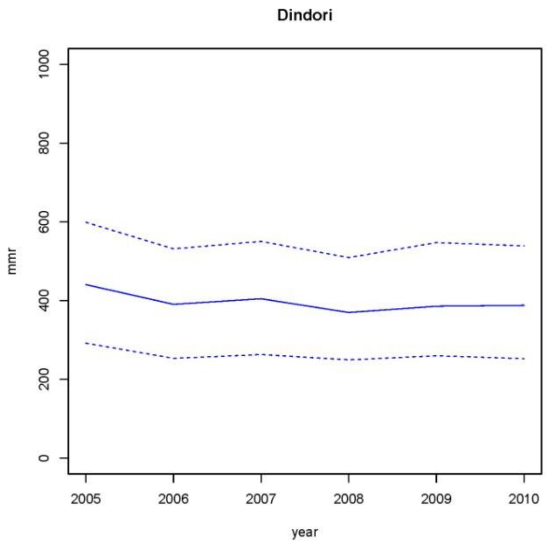
Dindori
mmr
year

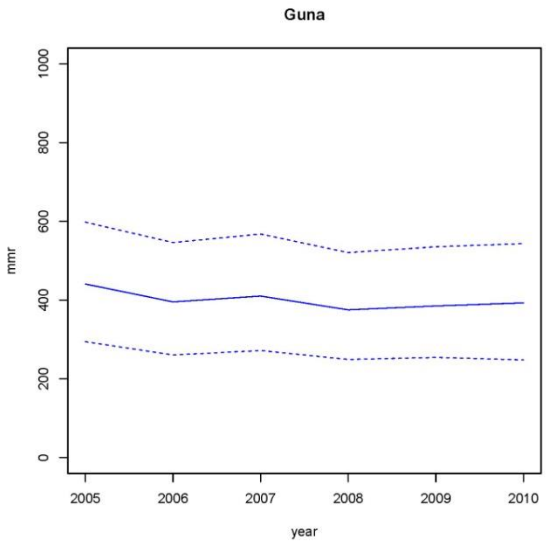
Guna
mmr
year

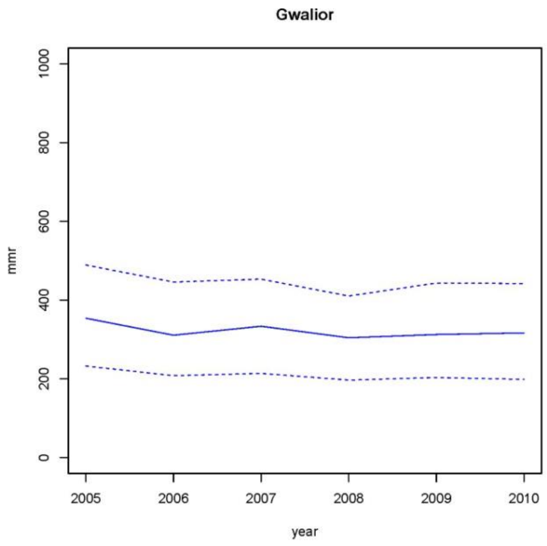
Gwalior
mmr
year

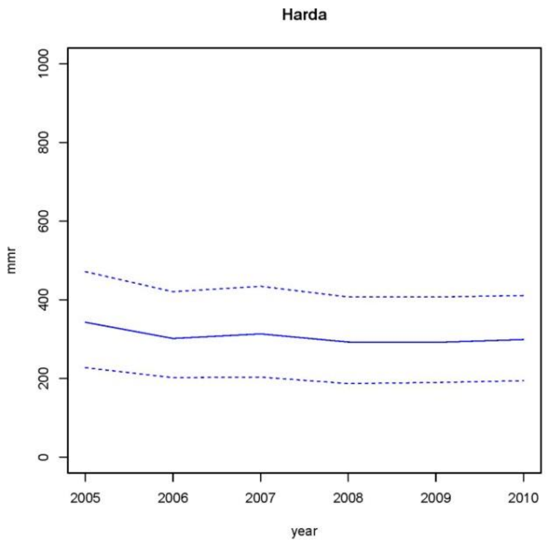
Harda
mmr
year

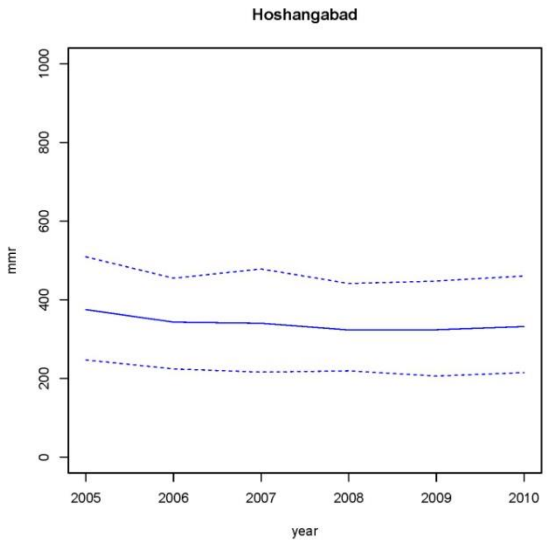
Hoshangabad
mmr
year

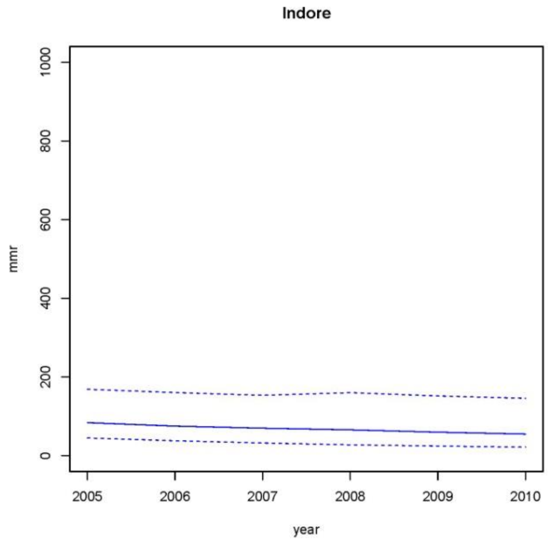
Indore
mmr
0
200
400
600
800
1000
2005
2006
2007
2008
2009
2010
year

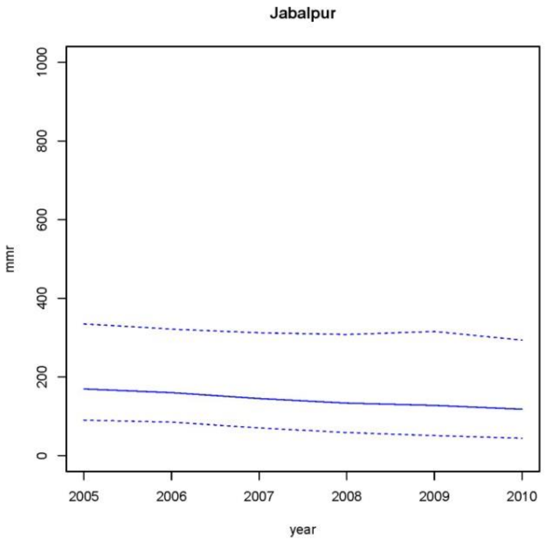
Jabalpur
mmr
0
200
400
600
800
1000
2005
2006
2007
2008
2009
2010
year

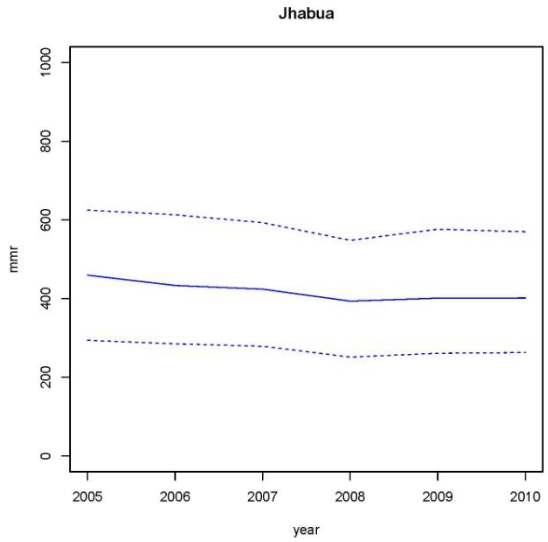
Jhabua
mmr
0
200
400
600
800
1000
2005
2006
2007
2008
2009
2010
year

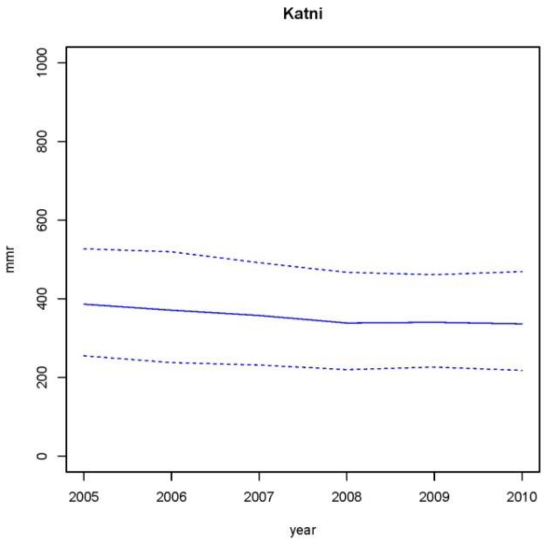
Katni
mmr
0
200
400
600
800
1000
2005
2006
2007
2008
2009
2010
year

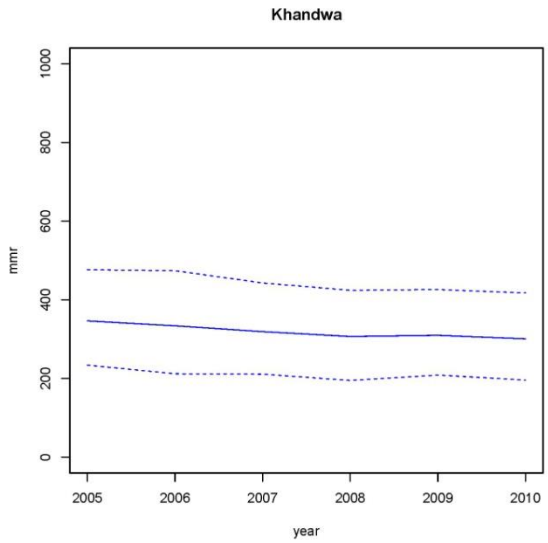
Khandwa
mmr
0
200
400
600
800
1000
2005
2006
2007
2008
2009
2010
year

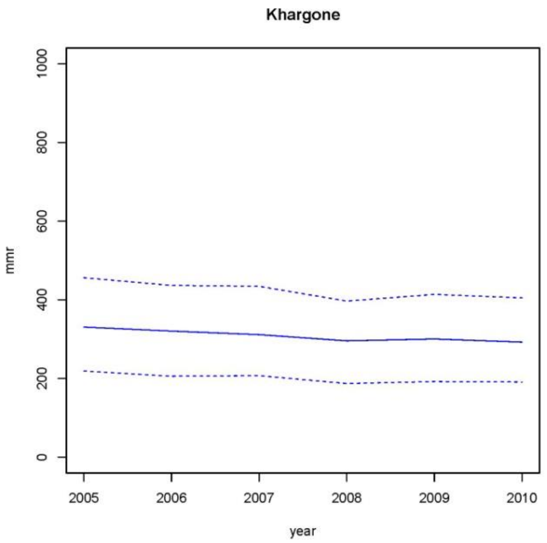
Khargone
mmr
0
200
400
600
800
1000
2005
2006
2007
2008
2009
2010
year

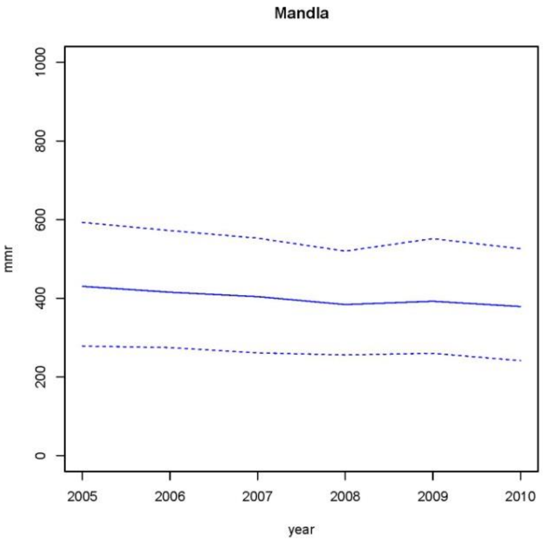
Mandla
mmr
0
200
400
600
800
1000
2005
2006
2007
2008
2009
2010
year

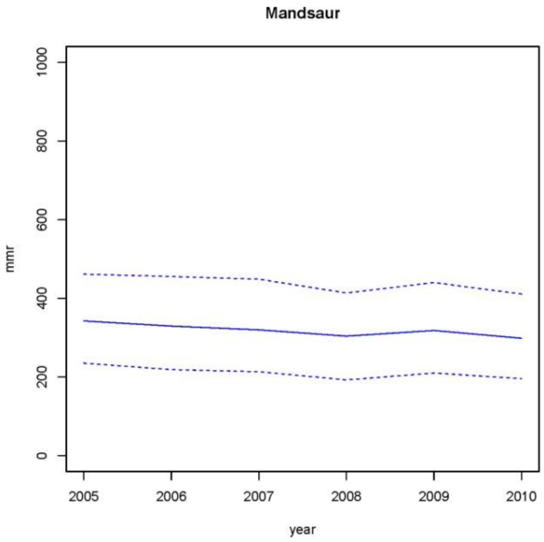
Mandsaur
mmr
0
200
400
600
800
1000
2005
2006
2007
2008
2009
2010
year

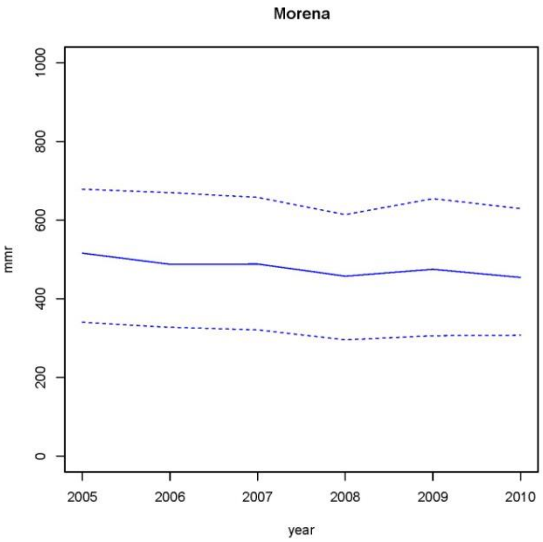
Morena
mmr
0
200
400
600
800
1000
2005
2006
2007
2008
2009
2010
year

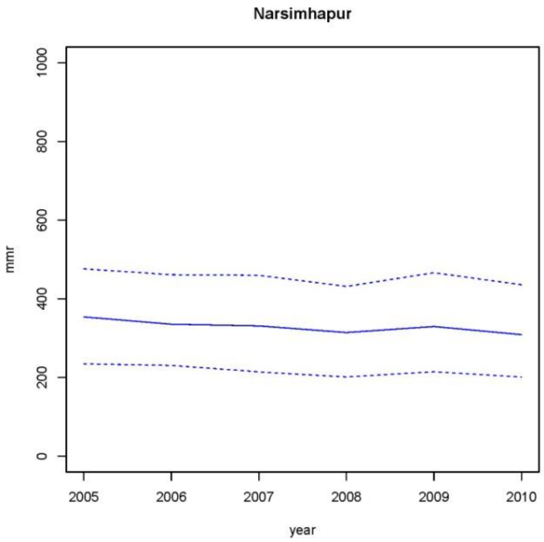
Narsimhapur
mmr
0
200
400
600
800
1000
2005
2006
2007
2008
2009
2010
year

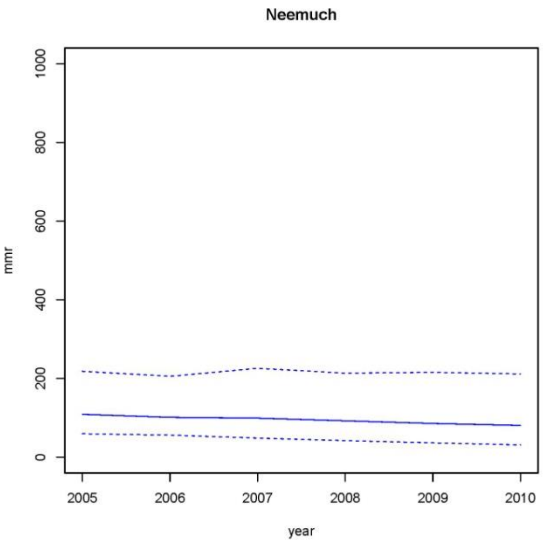
Neemuch
mmr
0
200
400
600
800
1000
2005
2006
2007
2008
2009
2010
year

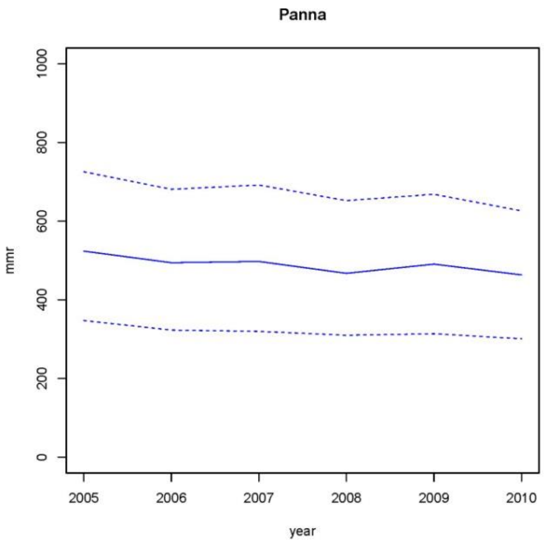
Panna
mmr
0
200
400
600
800
1000
2005
2006
2007
2008
2009
2010
year

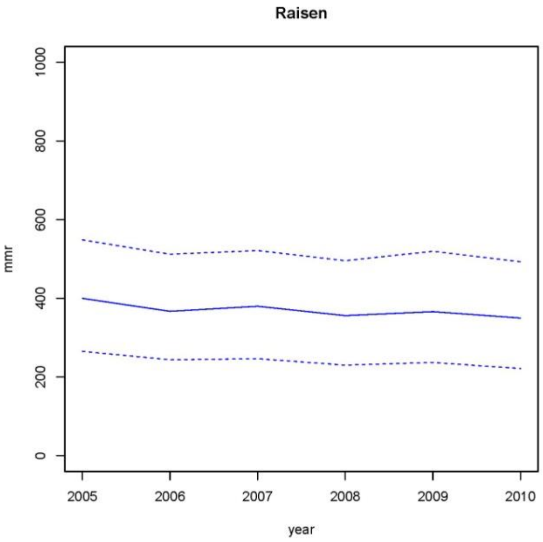
Raisen
mmr
0
200
400
600
800
1000
2005
2006
2007
2008
2009
2010
year
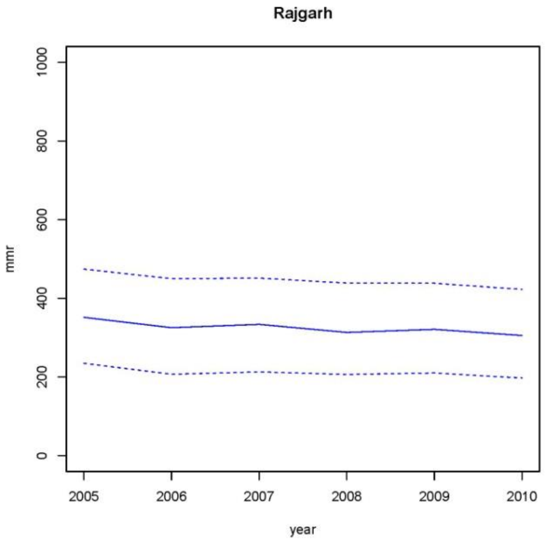
Rajgarh
mmr
0
200
400
600
800
1000
2005
2006
2007
2008
2009
2010
year
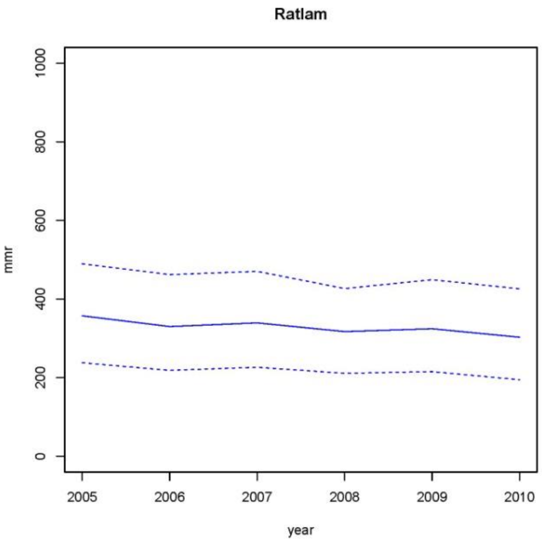
Ratlam
mmr
0
200
400
600
800
1000
2005
2006
2007
2008
2009
2010
year
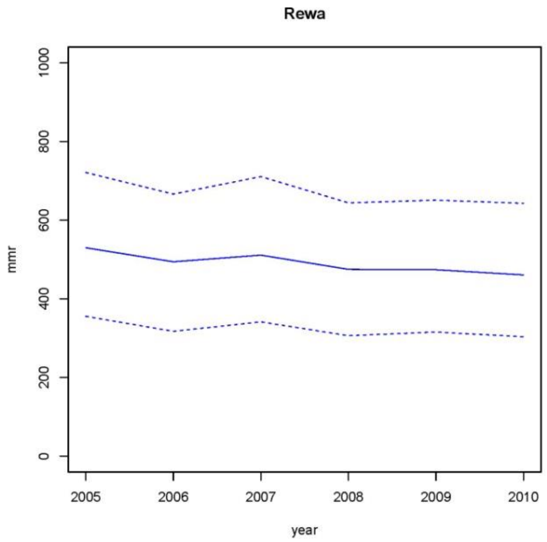
Rewa
mmr
0
200
400
600
800
1000
2005
2006
2007
2008
2009
2010
year
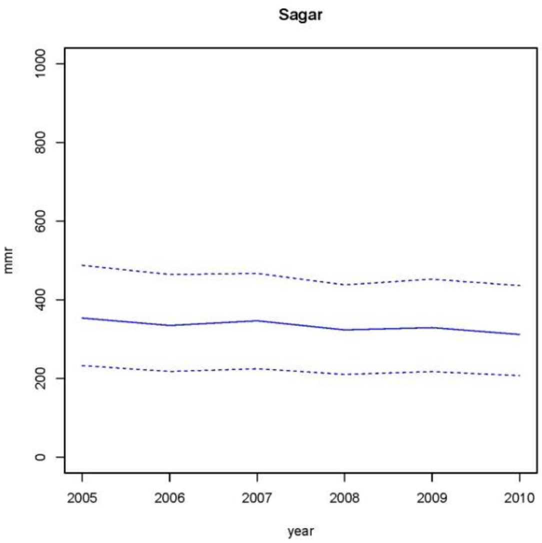
Sagar
mmr
0
200
400
600
800
1000
2005
2006
2007
2008
2009
2010
year
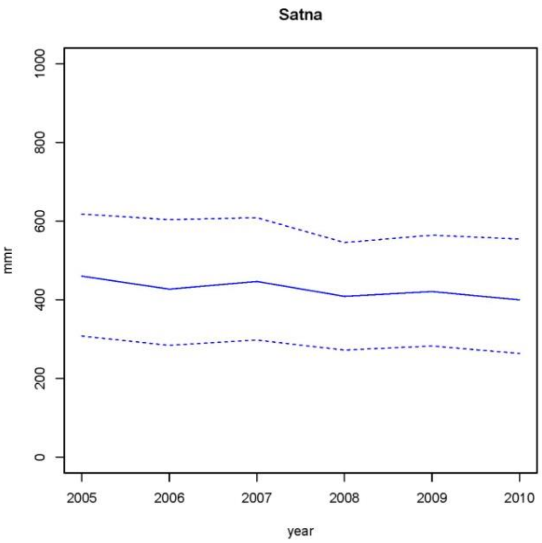
Satna
mmr
0
200
400
600
800
1000
2005
2006
2007
2008
2009
2010
year

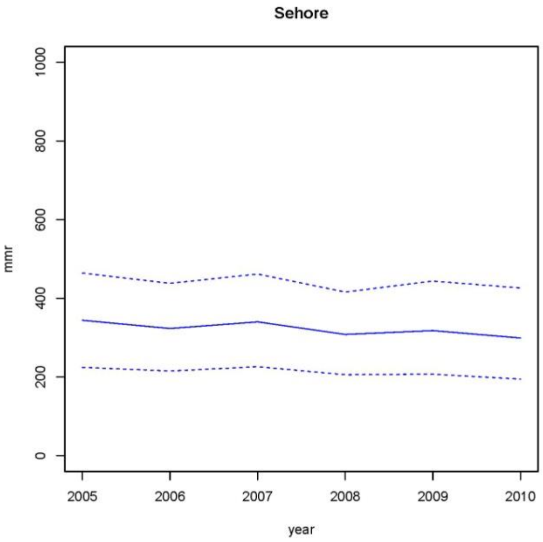
Sehore
mmr
0
200
400
600
800
1000
2005
2006
2007
2008
2009
2010
year

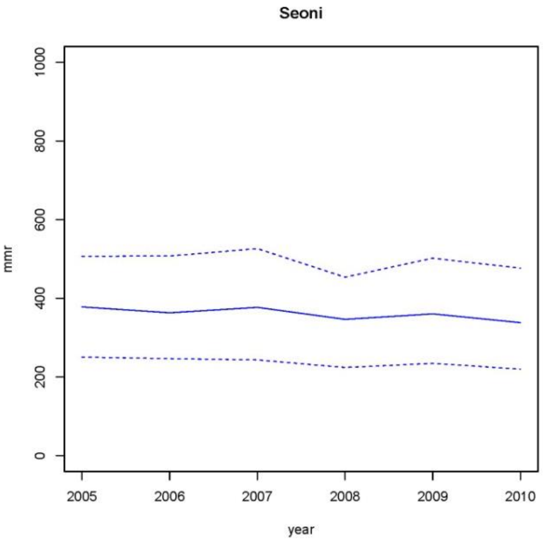
Seoni
mmr
0
200
400
600
800
1000
2005
2006
2007
2008
2009
2010
year

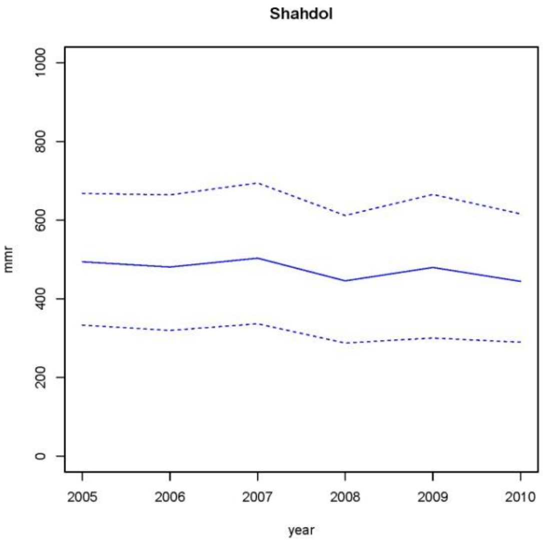
Shahdol
mmr
0
200
400
600
800
1000
2005
2006
2007
2008
2009
2010
year

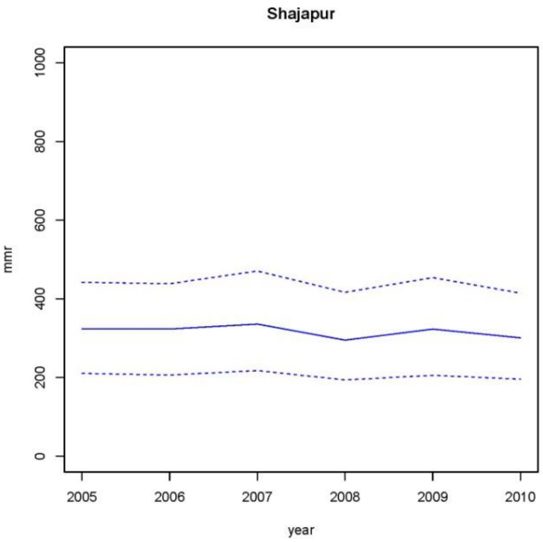
Shajapur
mmr
0
200
400
600
800
1000
2005
2006
2007
2008
2009
2010
year

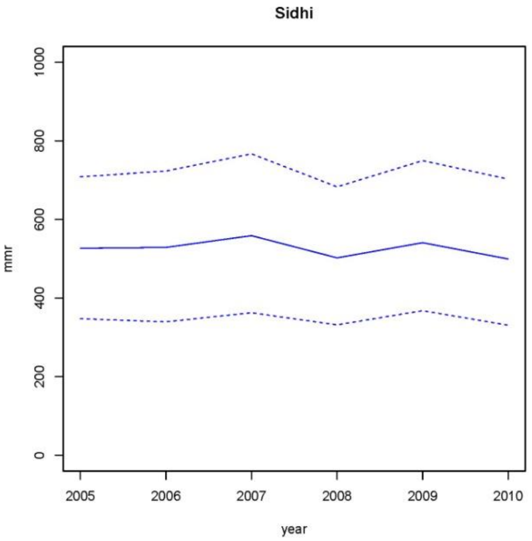
Sidhi
mmr
0
200
400
600
800
1000
2005
2006
2007
2008
2009
2010
year

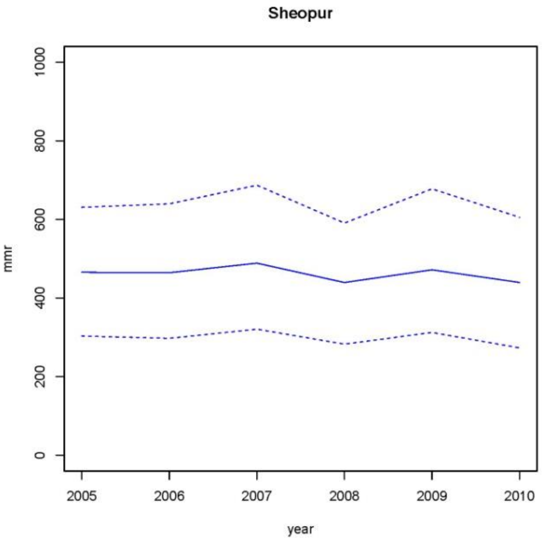
Sheopur
mmr
0
200
400
600
800
1000
2005
2006
2007
2008
2009
2010
year

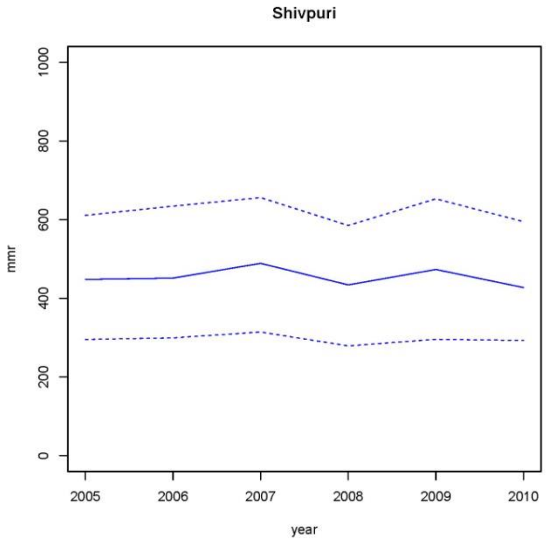
Shivpuri
mmr
0
200
400
600
800
1000
2005
2006
2007
2008
2009
2010
year

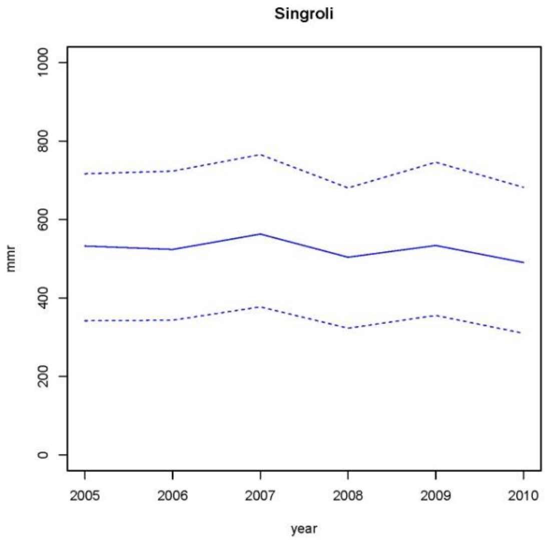
Singroli
mmr
0
200
400
600
800
1000
2005
2006
2007
2008
2009
2010
year

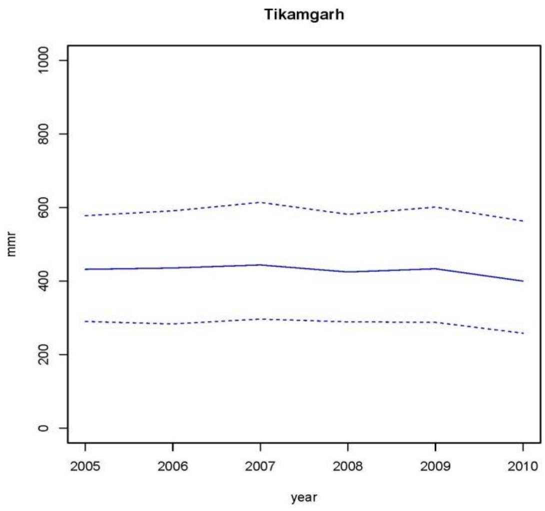
Tikamgarh
mmr
0
200
400
600
800
1000
2005
2006
2007
2008
2009
2010
year

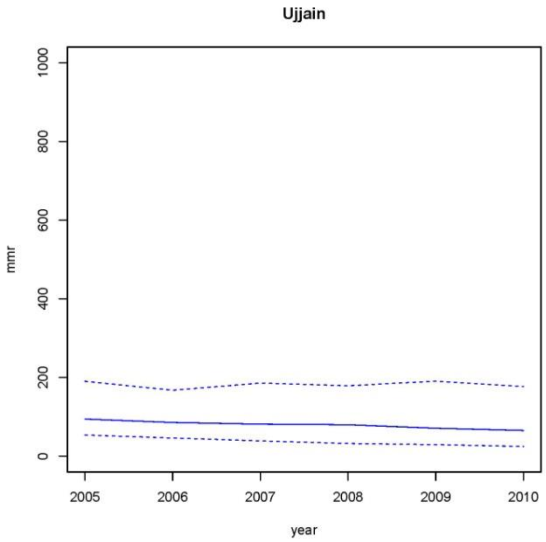
Ujjain
mmr
0
200
400
600
800
1000
2005
2006
2007
2008
2009
2010
year

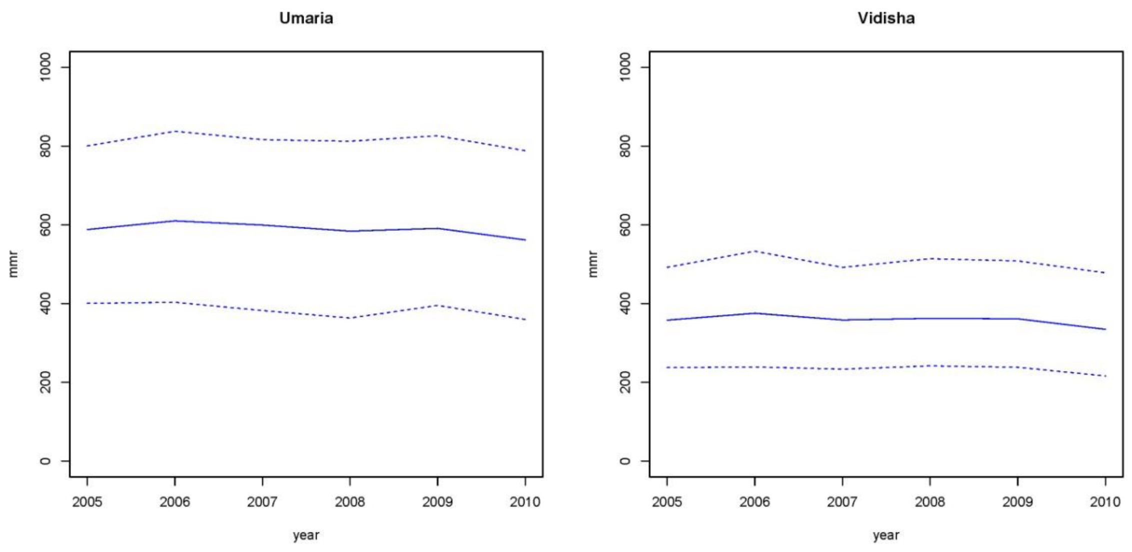

Webtable 3 Estimated MMR in 2010 in across 50 districts in Madhya Pradesh

District	MMR	CI	Rank
**Indore**	56	(22, 156)	1
**Ujjain**	66	(25, 180)	2
**Neemuch**	80	(31, 219)	3
**Bhopal**	100	(38, 293)	4
**Jabalpur**	114	(43, 315)	5
**Khargone**	289	(195, 404)	6
**Shajapur**	297	(198, 411)	7
**Mandsaur**	298	(193, 422)	8
**Khandwa**	302	(199, 422)	9
**Harda**	303	(201, 428)	10
**Sehore**	304	(195, 423)	11
**Rajgarh**	308	(197, 413)	12
**Dhar**	309	(197, 432)	13
**Ratlam**	309	(208, 427)	14
**Burhanpur**	312	(207, 423)	15
**Narsimhapur**	313	(209, 436)	16
**Sagar**	313	(205, 451)	17
**Gwalior**	318	(202, 439)	18
**Chhindwara**	322	(223, 453)	19
**Dewas**	329	(215, 462)	20
**Hoshangabad**	329	(212, 457)	21
**Datia**	335	(216, 464)	22
**Vidisha**	337	(220, 486)	23
**Seoni**	337	(228, 459)	24
**Katni**	338	(216, 468)	25

**Barwani**	338	(217, 470)	26
**Raisen**	346	(239, 480)	27
**Balaghat**	371	(249, 511)	28
**Mandla**	371	(256, 514)	29
**Betul**	374	(243, 519)	30
**Bhind**	383	(244, 526)	31
**Dindori**	388	(250, 549)	32
**Guna**	390	(256, 551)	33
**Ashoknagar**	392	(255, 547)	34
**Damoh**	399	(261, 551)	35
**Satna**	400	(265, 543)	36
**Jhabua**	401	(266, 560)	37
**Tikamgarh**	404	(261, 572)	38
**Alirajpur**	421	(272, 587)	39
**Shivpuri**	428	(285, 599)	40
**Chhatarpur**	433	(288, 606)	41
**Sheopur**	433	(287, 617)	42
**Shahdol**	442	(291, 633)	43
**Morena**	446	(287, 619)	44
**Rewa**	461	(300, 632)	45
**Panna**	464	(304, 659)	46
**Sidhi**	485	(323, 686)	47
**Anuppur**	485	(317, 674)	48
**Singroli**	492	(316, 676)	49
**Umaria**	554	(355, 792)	50
**Madhya Pradesh Overall**	327	(212, 474)	--

6. Percent changes in MMR from 2005 to 2010 across 50 districts in Madhya Pradesh

Webtable 4 Estimated percent changes in MMR with 95% confidence intervals

District	Percent Change in MMR (with 95% CI)
Alirajpur	-2.41 (-17.84, 16.81)
Anuppur	-1.94 (-18.08, 16.48)
Ashoknagar	-5.00 (-19.5, 16.71)
Balaghat	-3.29 (-18.95, 14.26)
Barwani	-6.19 (-21.01, 11.18)
Betul	-5.76 (-21.3, 12.29)
Bhind	-9.31 (-23.22, 6.88)
Bhopal	-20.13 (-46.41, 15.54)
Burhanpur	-9.35 (-24.9, 5.85)
Chhatarpur	-8.96 (-21.84, 8.78)
Chhindwara	-8.33 (-21.81, 8.08)
Damoh	-8.14 (-21.89, 11.16)
Datia	-10.62 (-24.42, 6.4)
Dewas	-10.21 (-25.15, 5.03)
Dhar	-10.87 (-26.17, 6.88)
Dindori	-11.13 (-26.03, 4.97)
Guna	-11.84 (-24.72, 4.05)
Gwalior	-10.7 (-25.12, 7.54)
Harda	-12.63 (-25.28, 3.19)
Hoshangabad	-11.78 (-25.11, 4.49)
Indore	-34.76 (-54.24, 0.5)
Jabalpur	-32.54 (-54.14,-2.82)
Jhabua	-11.57 (-26.14, 5.59)
Katni	-13.13 (-27.9, 3.17)
Khandwa	-10.85 (-25.34, 4.78)
Khargone	-12.88 (-26.83, 2.6)
Mandla	-14.1 (-27.89, 1.55)
Mandsaur	-11.78 (-26.07, 5.27)
Morena	-12.54 (-27.28, 2.66)
Narsimhapur	-10.78 (-24.52, 6.29)
Neemuch	-26.64 (-50.08, 7.3)
Panna	-10.88 (-24.59, 6.22)
Raisen	-13.41 (-27.51, 4.34)
Rajgarh	-11.85 (-26.24, 4.92)
Ratlam	-11.59 (-26.75, 4.58)
Rewa	-12.88 (-25.49, 3.97)
Sagar	-12.64 (-26.17, 5.3)

**Satna**	-12.03 (-25.34, 3.68)
**Sehore**	-11.07 (-26.09, 5.45)
**Seoni**	-11.42 (-24.45, 4.65)
**Shahdol**	-11.49 (-25.08, 5.27)
**Shajapur**	-8.6 (-23.54, 10.62)
**Sheopur**	-5.49 (-20.97, 12.27)
**Shivpuri**	-4.63 (-19.54, 13.03)
**Sidhi**	-7.79 (-21.73, 12.03)
**Singroli**	-7.29 (-21.68, 10.55)
**Tikamgarh**	-6.84 (-21.56, 11.72)
**Ujjain**	-27.56 (-50.65, 3.34)
**Umaria**	-3.5 (-20.63, 16.36)
**Vidisha**	-5.78 (-21.73, 13.99)
**Madhya Pradesh Overall**	-10.88 (-0.18, -0.03)

Webappendix II Estimation of the impact of JSY

1. **Data**

Estimation of Total delivery

The number of institutional delivery reported in the Health Bulletin reports were adjusted by a correction factor derived based on DLHS-3. The values are shown in web table 3.

Webtable 5 Adjustment factor for district level intuitional delivery based on percent of delivery estimated by Health Bulletin and DLHS-3 in 2007.

District	Adjustment factor
Alirajpur	0.48
Anuppur	0.48
Ashoknagar	0.48
Balaghat	0.68
Barwani	0.43
Betul	0.59
Bhind	0.79
Bhopal	0.66
Burhanpur	0.48
Chhatarpur	0.67
Chhindwara	0.57
Damoh	0.57
Datia	0.57
Dewas	0.75
Dhar	0.75
Dindori	0.34
Guna	0.53
Gwalior	0.90
Harda	0.97
Hoshangabad	0.77
Indore	0.85
Jabalpur	0.83
Jhabua	0.64
Katni	0.51
Khandwa	0.48
Khargone	0.48
Mandla	0.55
Mandsaur	0.48
Morena	0.77
Narsimhapur	0.48
Neemuch	0.72
Panna	0.50
Raisen	0.52

**Rajgarh**	0.74
**Ratlam**	0.68
**Rewa**	0.72
**Sagar**	0.83
**Satna**	0.59
**Sehore**	0.74
**Seoni**	0.81
**Shahdol**	0.48
**Shajapur**	0.81
**Sheopur**	0.58
**Shivpuri**	0.62
**Sidhi**	0.49
**Singroli**	0.48
**Tikamgarh**	0.80
**Ujjain**	0.97
**Umaria**	0.48
**Vidisha**	0.67

2. Changes in MMR and changes in the proportion of institutional deliveries (JSY-supported and non-JSY institutional deliveries) from 2005 to 2010.

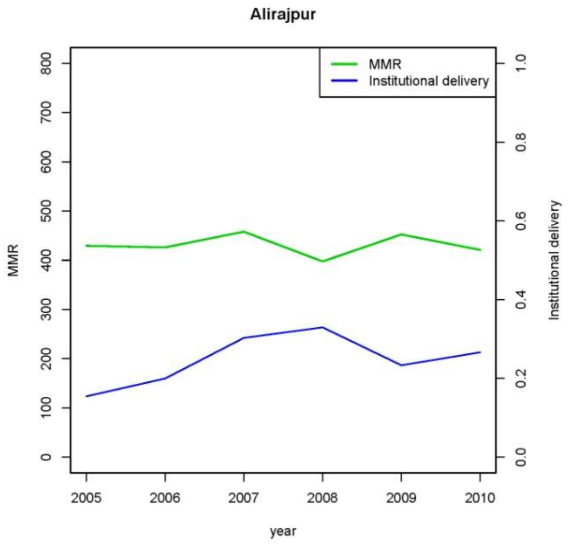

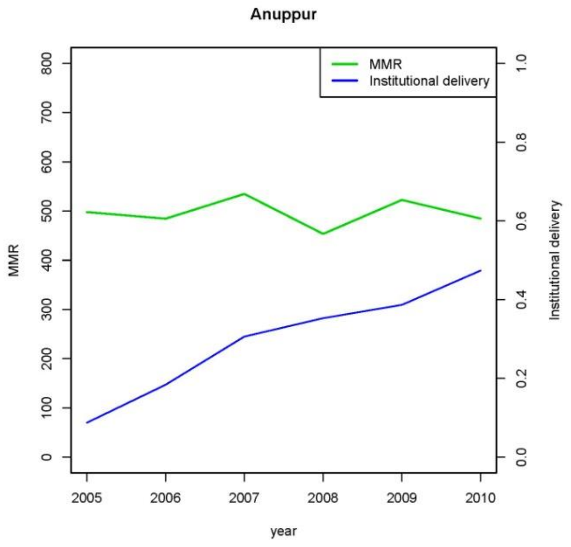

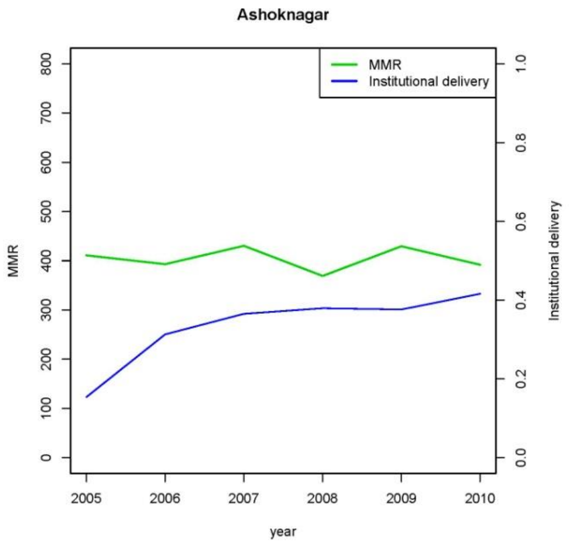

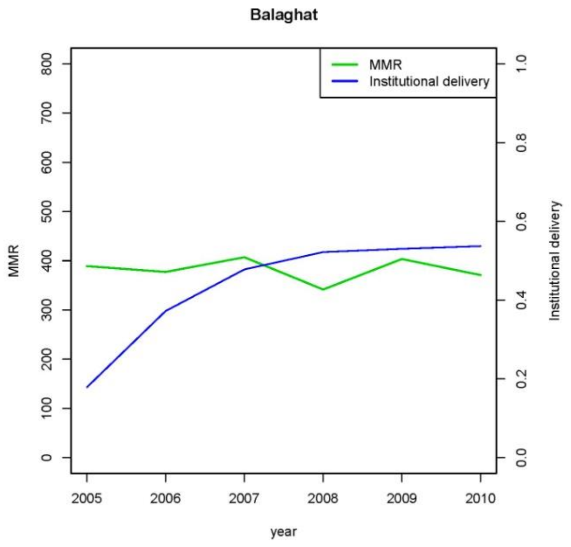

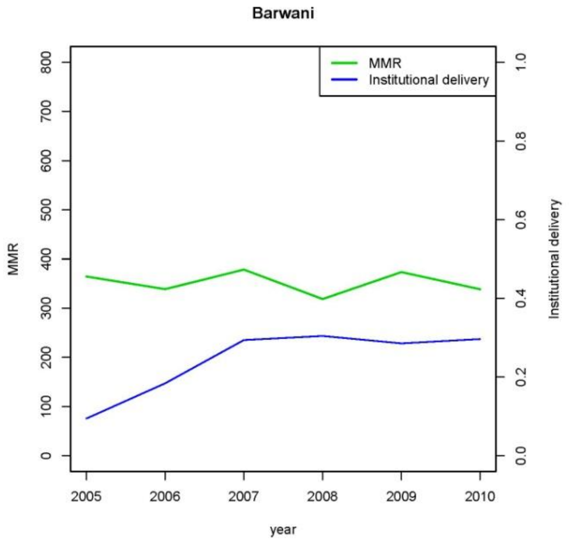
Barwani
MMR
Institutional delivery
year

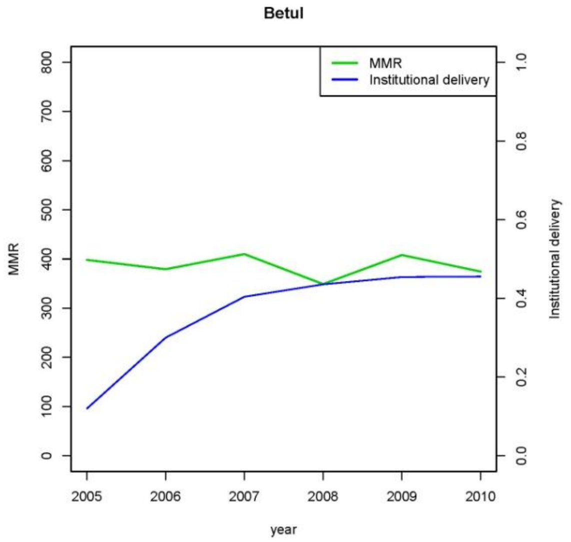
Betul
MMR
Institutional delivery
year

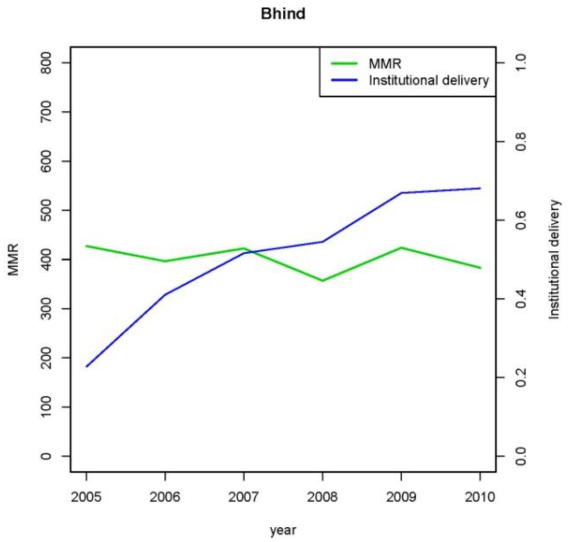
Bhind
MMR
Institutional delivery
year

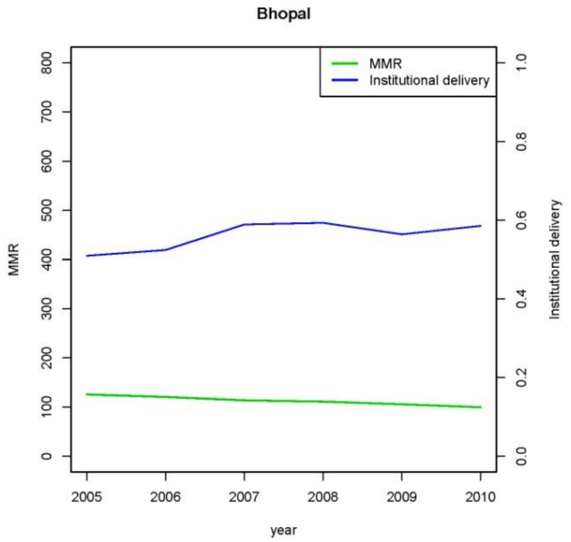
Bhopal
MMR
Institutional delivery
year

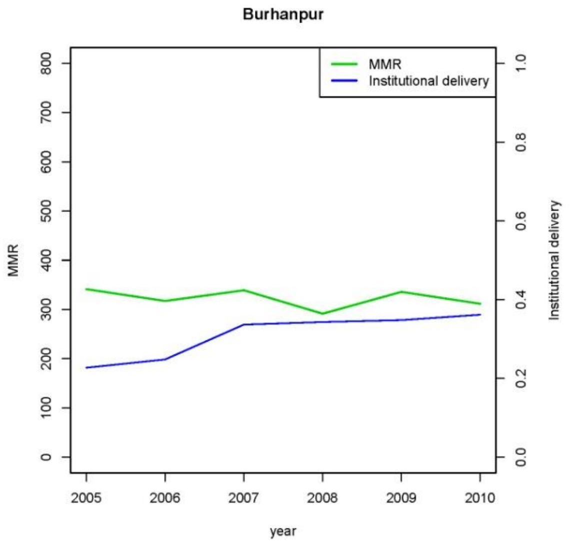
Burhanpur
MMR
Institutional delivery
year

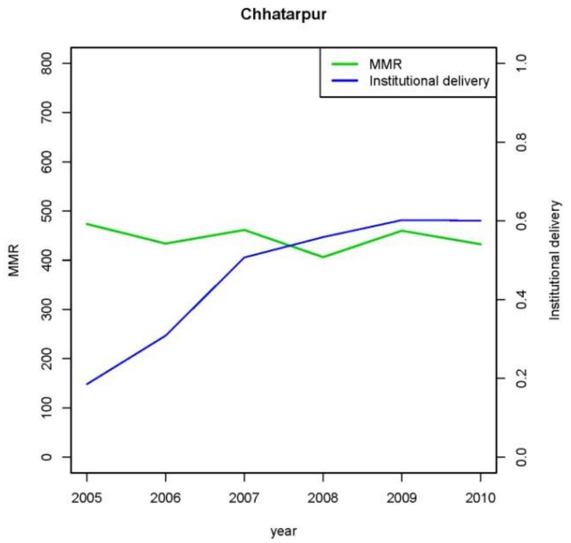
Chhatarpur
MMR
Institutional delivery
year

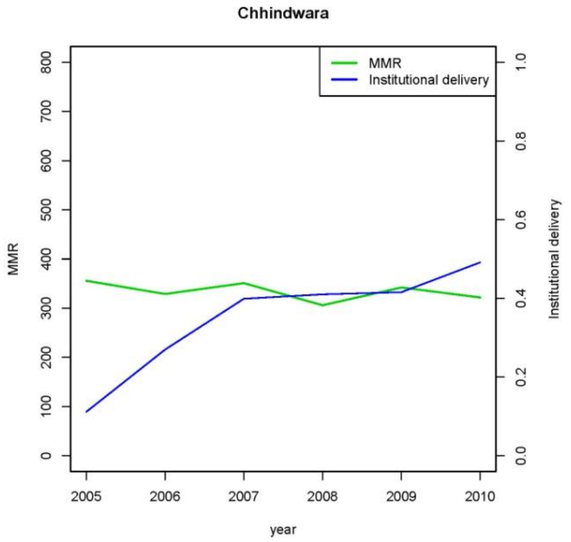
Chhindwara
MMR
Institutional delivery
year

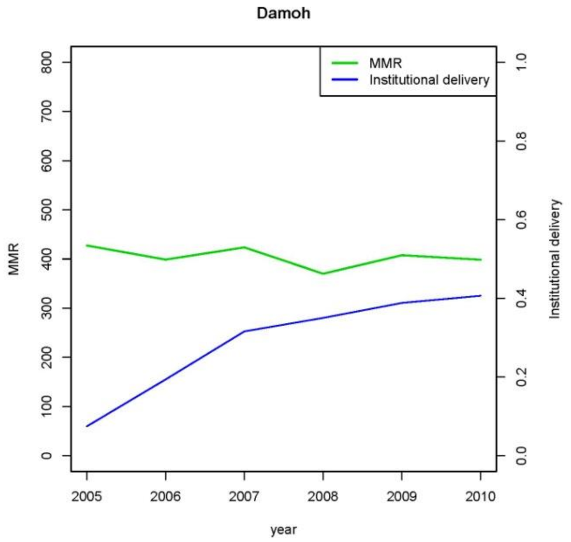
Damoh
MMR
Institutional delivery
year

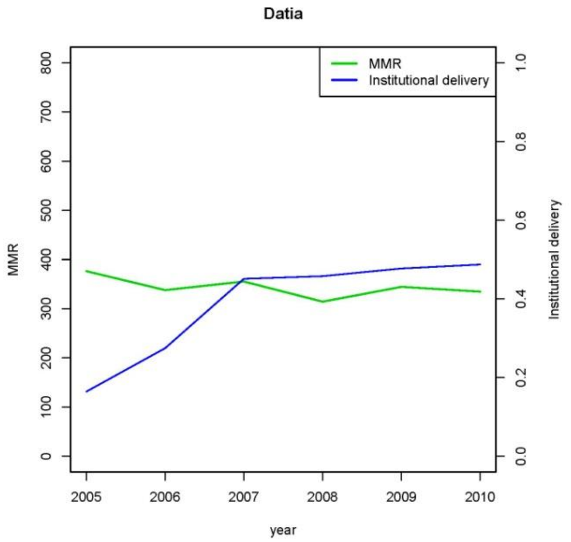
Datia
MMR
Institutional delivery
year

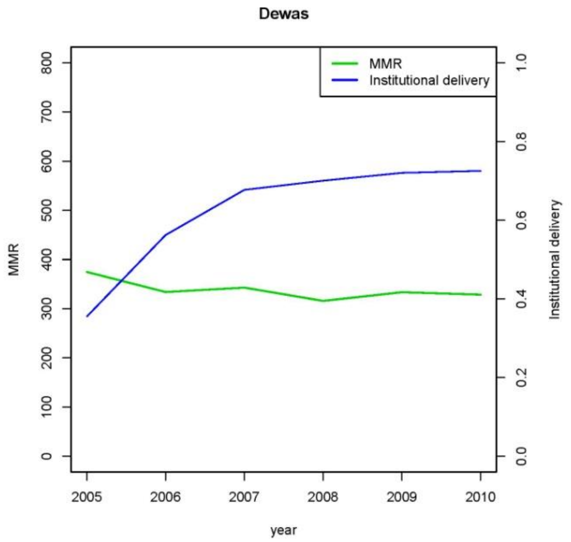
Dewas
MMR
Institutional delivery
year

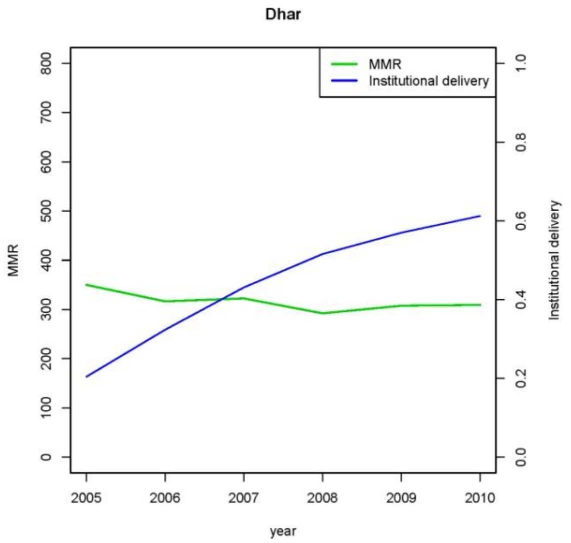
Dhar
MMR
Institutional delivery
year

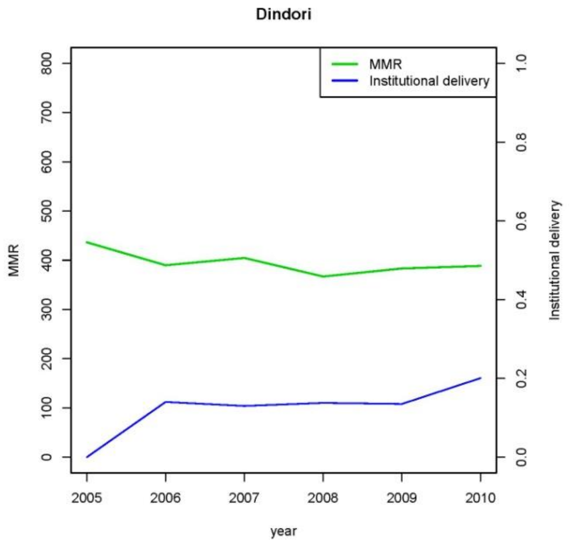
Dindori
MMR
Institutional delivery
year

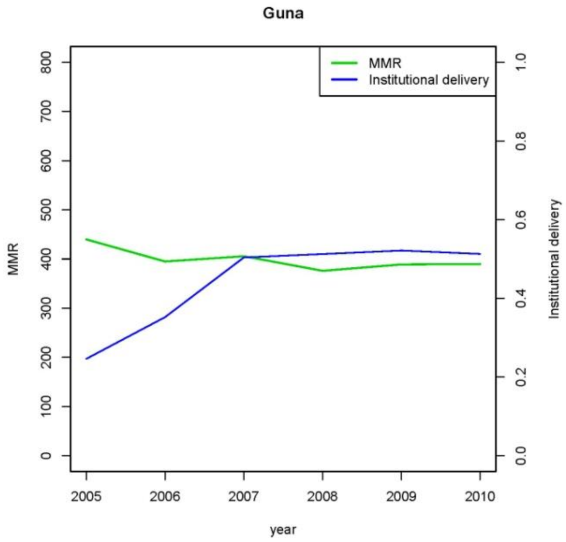
Guna
MMR
Institutional delivery
MMR
Institutional delivery
year

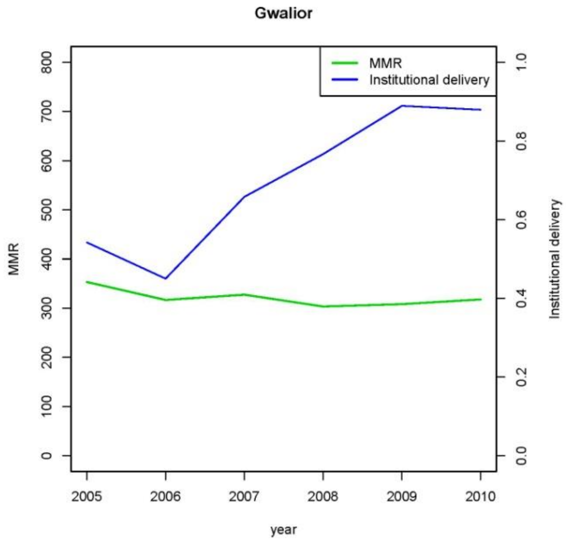
Gwalior
MMR
Institutional delivery
MMR
Institutional delivery
year

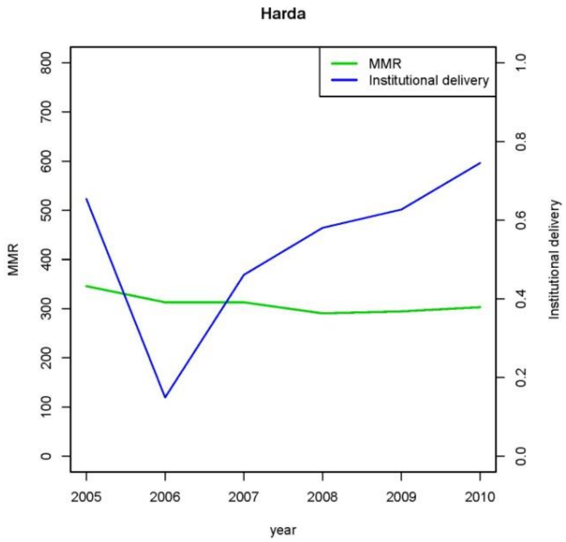
Harda
MMR
Institutional delivery
MMR
Institutional delivery
year

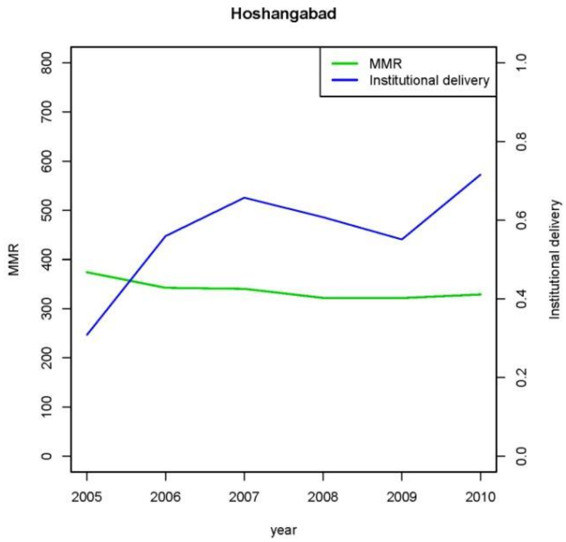
Hoshangabad
MMR
Institutional delivery
MMR
Institutional delivery
year

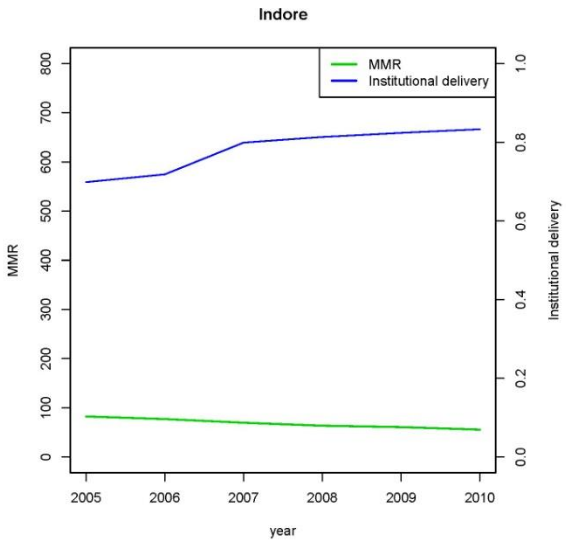
Indore
MMR
Institutional delivery
MMR
Institutional delivery
year

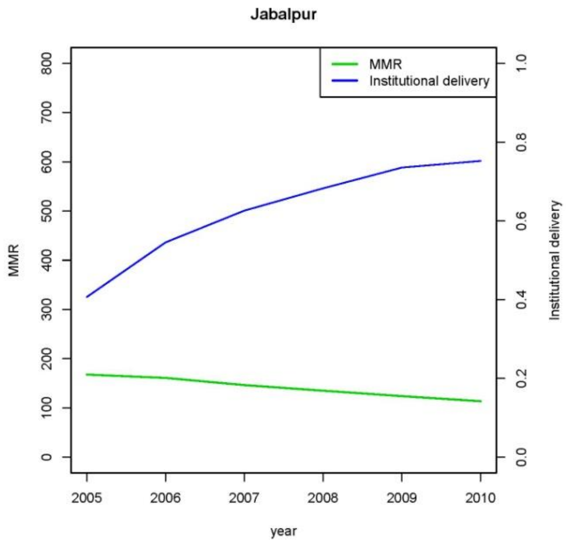
Jabalpur
MMR
Institutional delivery
MMR
Institutional delivery
year

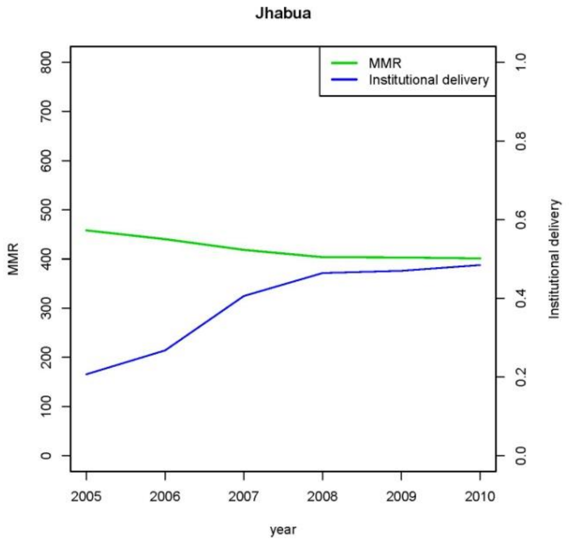
Jhabua
MMR
Institutional delivery
year
MMR
Institutional delivery

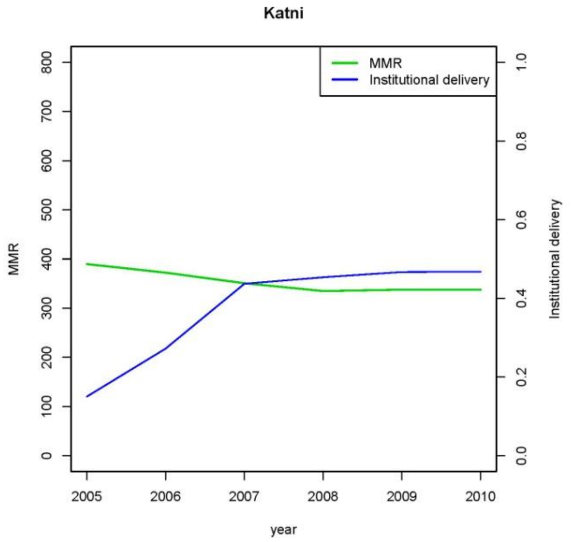
Katni
MMR
Institutional delivery
year
MMR
Institutional delivery

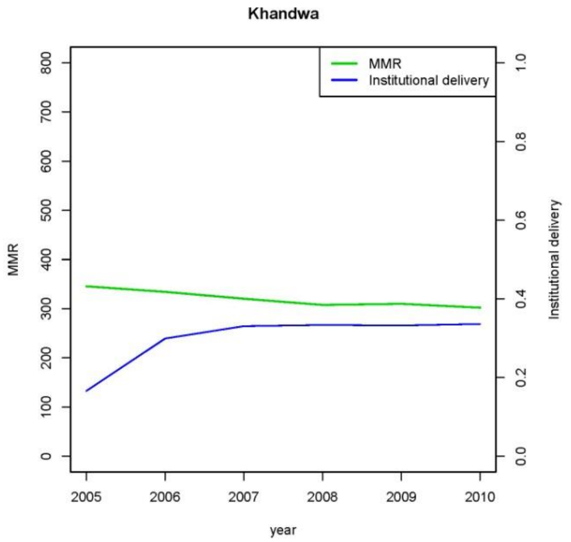
Khandwa
MMR
Institutional delivery
year
MMR
Institutional delivery

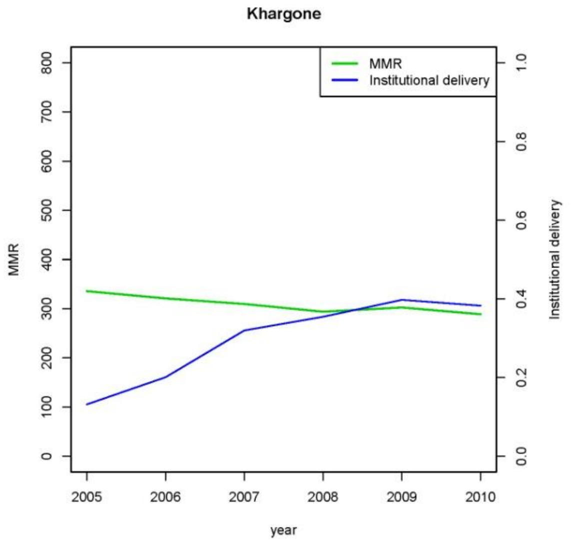
Khargone
MMR
Institutional delivery
year
MMR
Institutional delivery

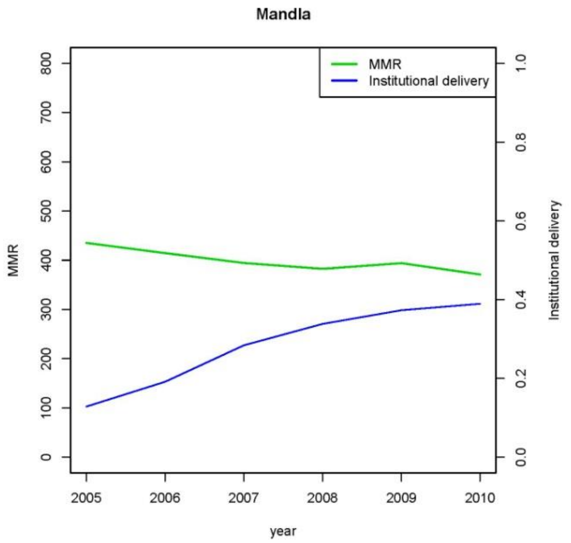
Mandla
MMR
Institutional delivery
year
MMR
Institutional delivery

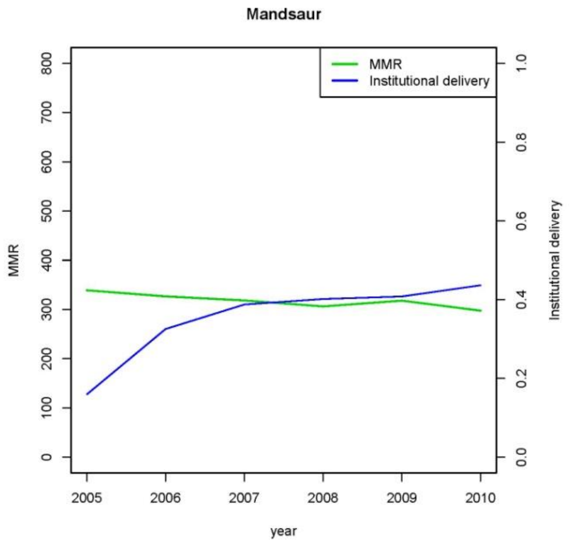
Mandsaur
MMR
Institutional delivery
year
MMR
Institutional delivery

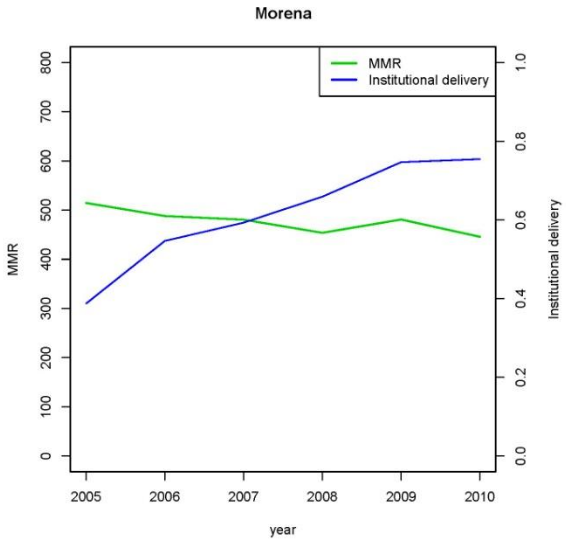
Morena
MMR
Institutional delivery
0 100 200 300 400 500 600 700 800
0.0 0.2 0.4 0.6 0.8 1.0
2005 2006 2007 2008 2009 2010
year

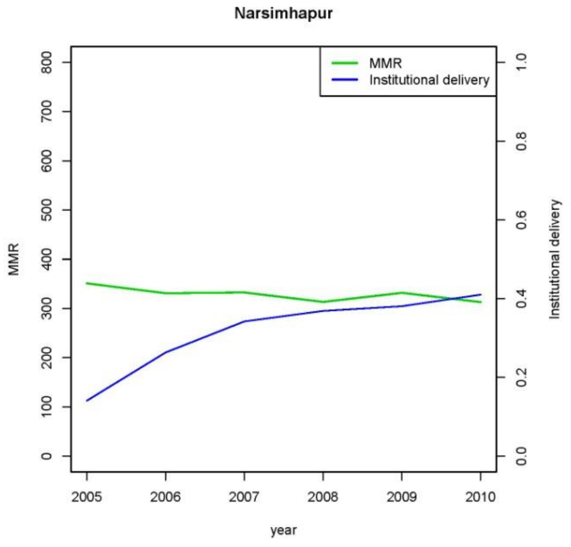
Narsimhapur
MMR
Institutional delivery
0 100 200 300 400 500 600 700 800
0.0 0.2 0.4 0.6 0.8 1.0
2005 2006 2007 2008 2009 2010
year

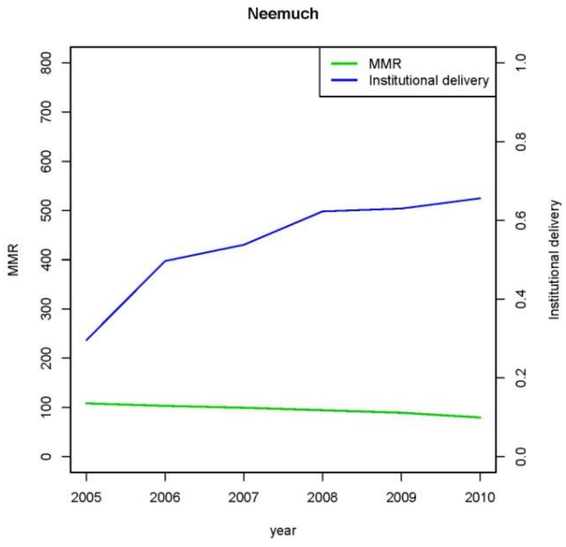
Neemuch
MMR
Institutional delivery
0 100 200 300 400 500 600 700 800
0.0 0.2 0.4 0.6 0.8 1.0
2005 2006 2007 2008 2009 2010
year

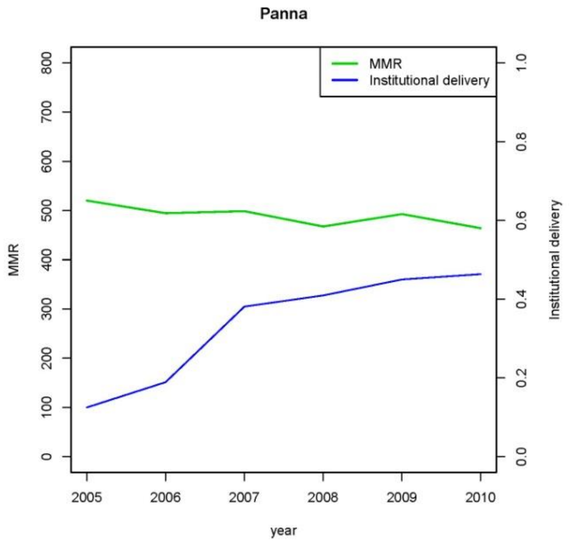
Panna
MMR
Institutional delivery
0 100 200 300 400 500 600 700 800
0.0 0.2 0.4 0.6 0.8 1.0
2005 2006 2007 2008 2009 2010
year

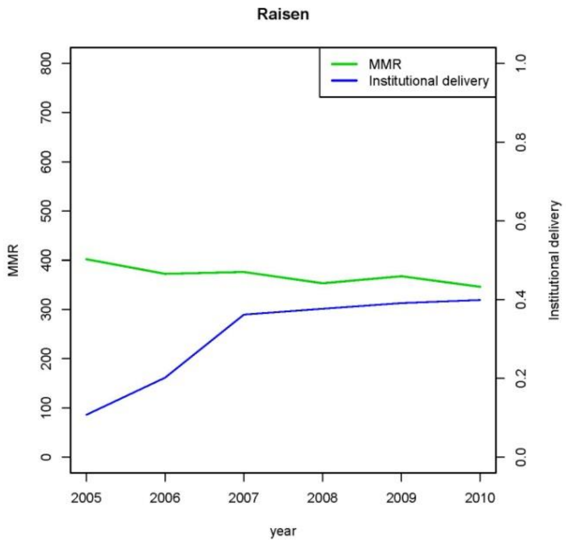
Raisen
MMR
Institutional delivery
0 100 200 300 400 500 600 700 800
0.0 0.2 0.4 0.6 0.8 1.0
2005 2006 2007 2008 2009 2010
year

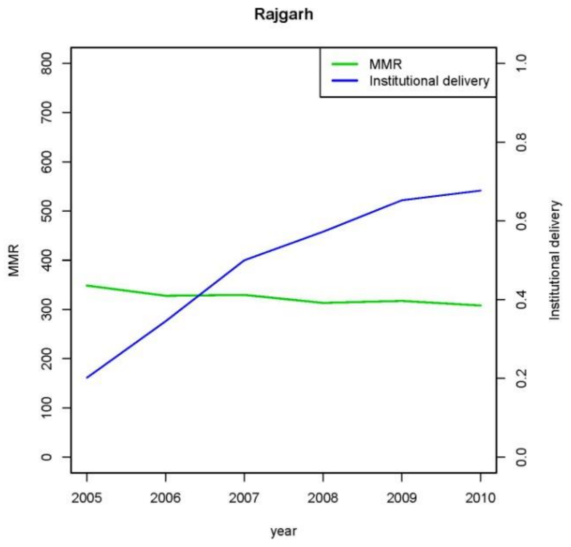
Rajgarh
MMR
Institutional delivery
0 100 200 300 400 500 600 700 800
0.0 0.2 0.4 0.6 0.8 1.0
2005 2006 2007 2008 2009 2010
year

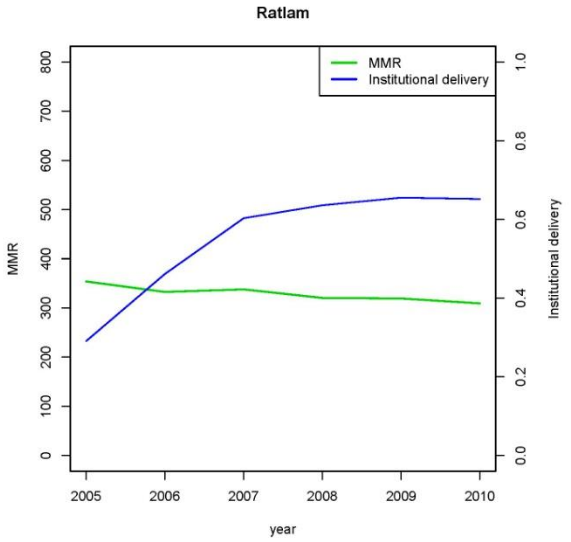
Ratlam
MMR
Institutional delivery
year
MMR
Institutional delivery

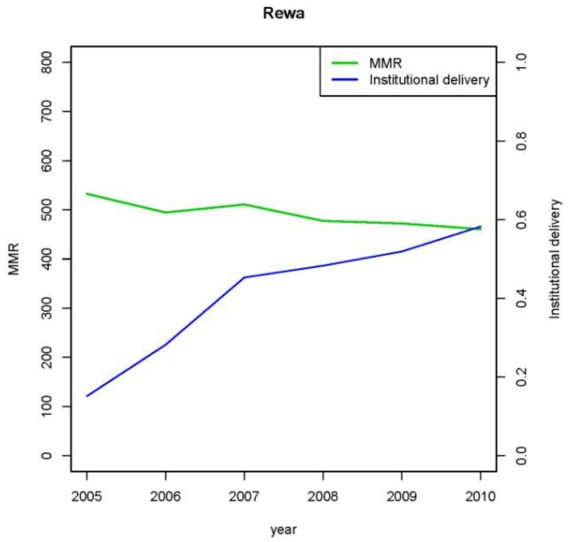
Rewa
MMR
Institutional delivery
year
MMR
Institutional delivery

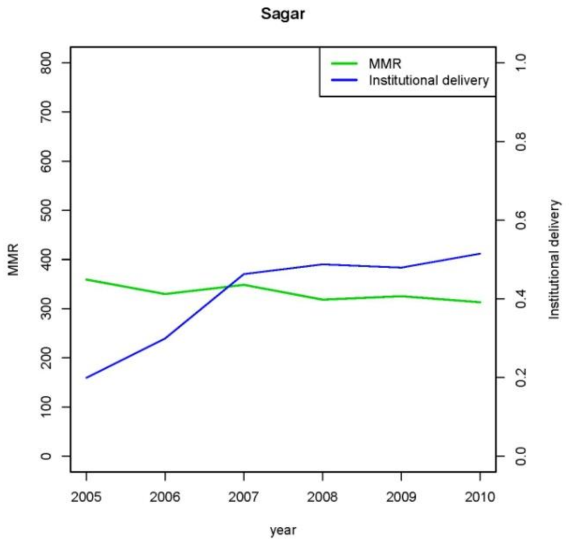
Sagar
MMR
Institutional delivery
year
MMR
Institutional delivery

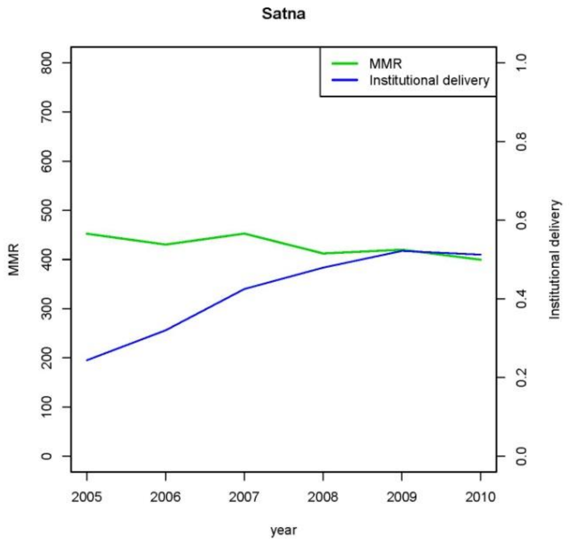
Satna
MMR
Institutional delivery
year
MMR
Institutional delivery

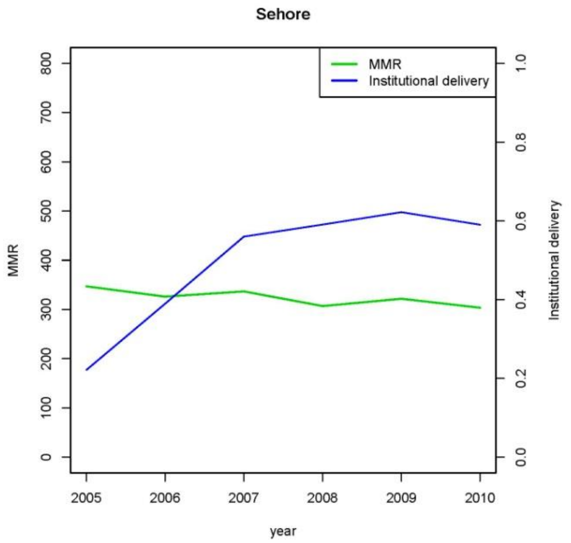
Sehore
MMR
Institutional delivery
year
MMR
Institutional delivery

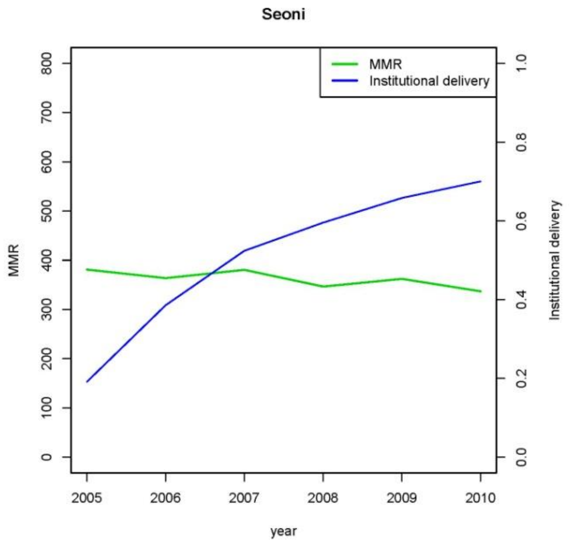
Seoni
MMR
Institutional delivery
year
MMR
Institutional delivery

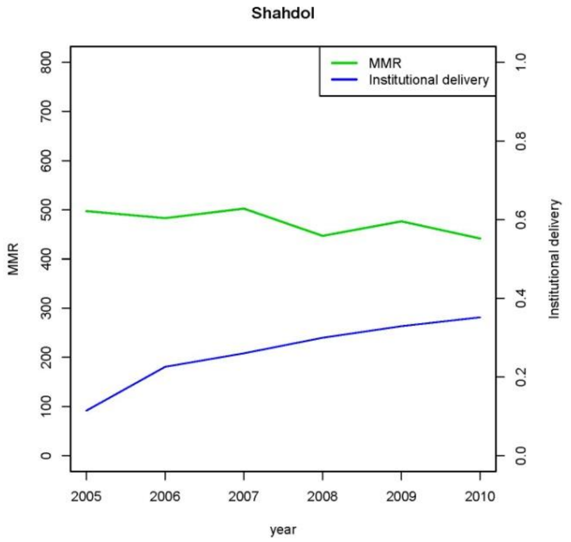
Shahdol
MMR
Institutional delivery
year
MMR
Institutional delivery
0 100 200 300 400 500 600 700 800
0.0 0.2 0.4 0.6 0.8 1.0
2005 2006 2007 2008 2009 2010

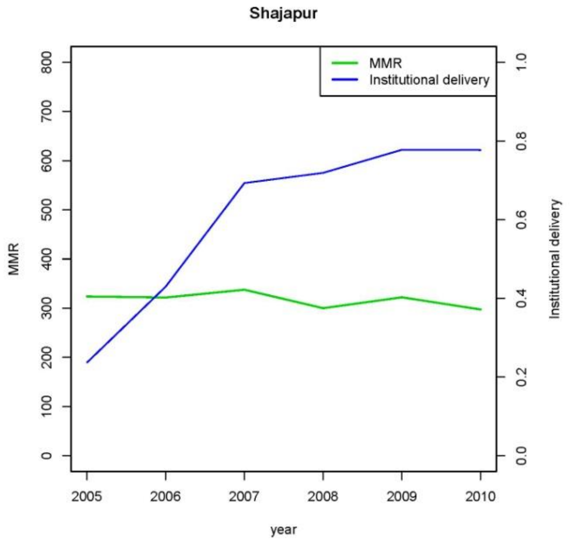
Shajapur
MMR
Institutional delivery
year
MMR
Institutional delivery
0 100 200 300 400 500 600 700 800
0.0 0.2 0.4 0.6 0.8 1.0
2005 2006 2007 2008 2009 2010

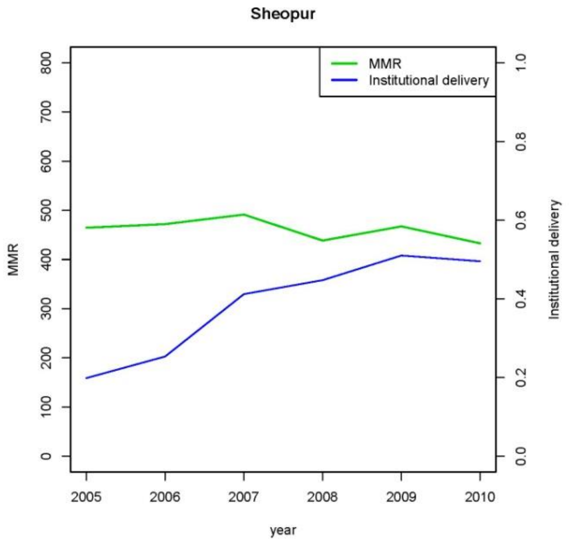
Sheopur
MMR
Institutional delivery
year
MMR
Institutional delivery
0 100 200 300 400 500 600 700 800
0.0 0.2 0.4 0.6 0.8 1.0
2005 2006 2007 2008 2009 2010

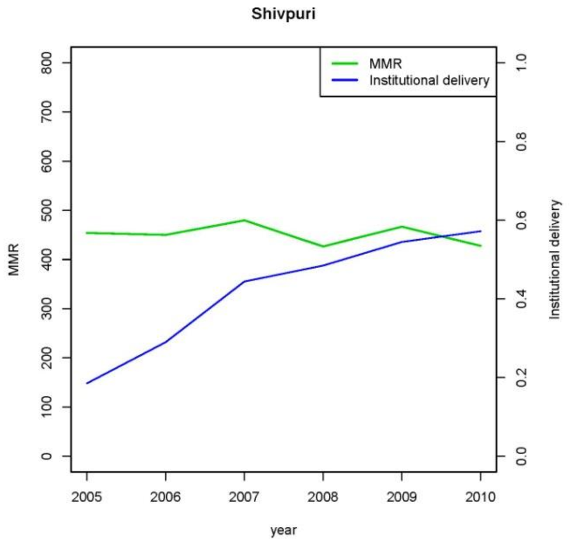
Shivpuri
MMR
Institutional delivery
year
MMR
Institutional delivery
0 100 200 300 400 500 600 700 800
0.0 0.2 0.4 0.6 0.8 1.0
2005 2006 2007 2008 2009 2010

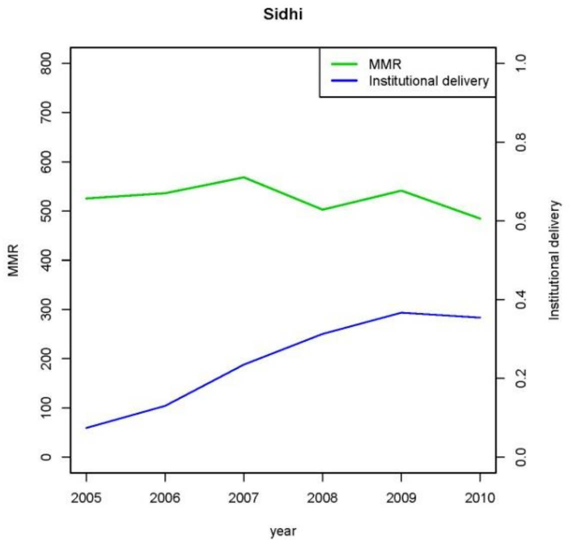
Sidhi
MMR
Institutional delivery
year
MMR
Institutional delivery
0 100 200 300 400 500 600 700 800
0.0 0.2 0.4 0.6 0.8 1.0
2005 2006 2007 2008 2009 2010

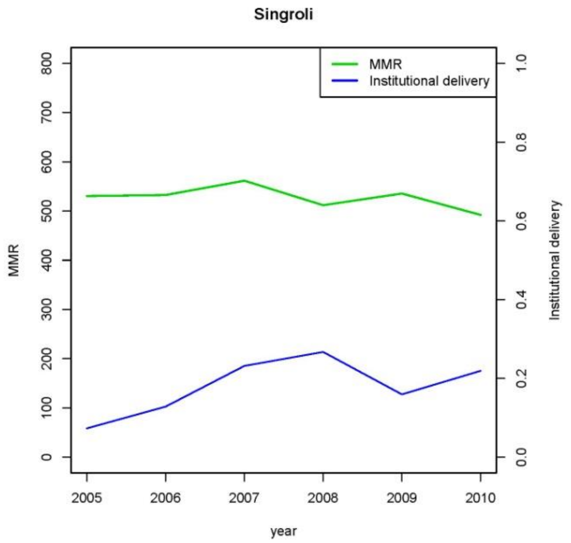
Singroli
MMR
Institutional delivery
year
MMR
Institutional delivery
0 100 200 300 400 500 600 700 800
0.0 0.2 0.4 0.6 0.8 1.0
2005 2006 2007 2008 2009 2010

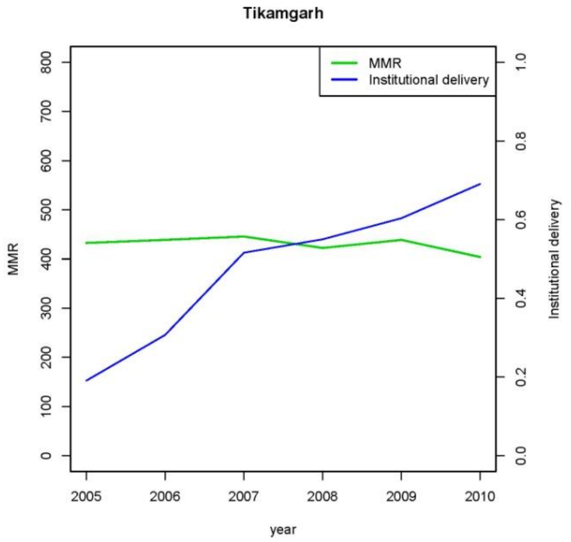
Tikamgarh
MMR
Institutional delivery
0 100 200 300 400 500 600 700 800
0.0 0.2 0.4 0.6 0.8 1.0
2005 2006 2007 2008 2009 2010
year

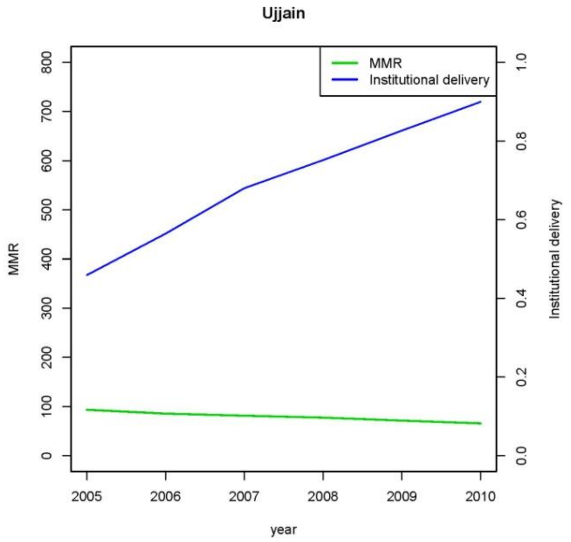
Ujjain
MMR
Institutional delivery
0 100 200 300 400 500 600 700 800
0.0 0.2 0.4 0.6 0.8 1.0
2005 2006 2007 2008 2009 2010
year

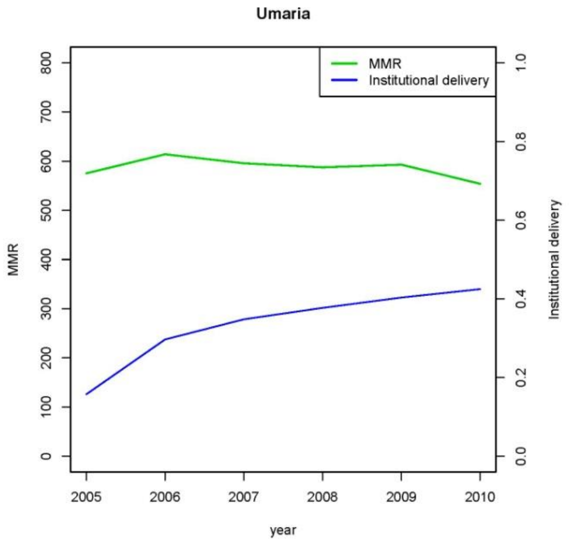
Umaria
MMR
Institutional delivery
0 100 200 300 400 500 600 700 800
0.0 0.2 0.4 0.6 0.8 1.0
2005 2006 2007 2008 2009 2010
year

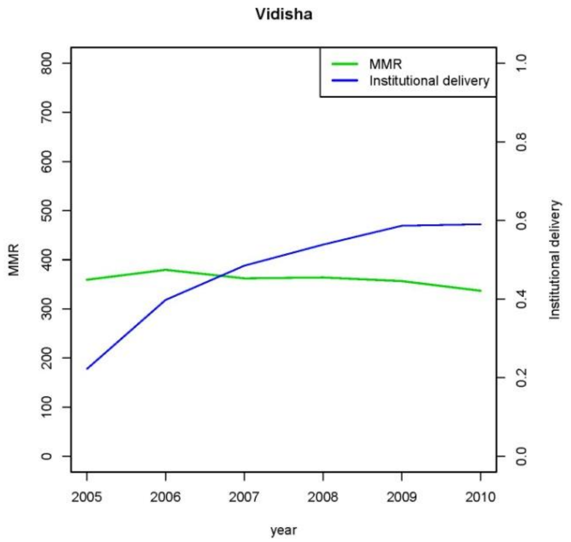
Vidisha
MMR
Institutional delivery
0 100 200 300 400 500 600 700 800
0.0 0.2 0.4 0.6 0.8 1.0
2005 2006 2007 2008 2009 2010
year

3. Alternative models

We consider a simpler model were we assumed that JSY has an uniform impact across all districts. With regard to the association between MMR and JSY-supported institutional deliveries, the following model was considered.

$$\log(MMR_{d,t}) = \beta_0 + \beta_1 Urban_d + \beta_2 Lit_d + \beta_3 ANC3_{d,t} + \beta_4 NJSY_{d,t} + \beta_5 JSY_{d,t} + \alpha_t + \eta_d$$

Note that the district-specific slope on $JSY_{d,t}$ is omitted. The coefficient estimates are presented in Webtable 6. Consistent with the previous analysis, no significant relationship was found between JSY-supported delivery and MMR across the districts.

Webtable 6 Estimated fixed effect coefficients from the multilevel regression model without district-specific random slopes.

	Estimated Coefficients	CI	P-values
**Intercept**	6.692	(6.541,6.841)	0
**Literacy**	-0.002	(-0.005, 0.007)	0.142
**Urban**	-0.013	(-0.017,-0.010)	0
**ANC3**	-0.009	(-0.011, -0.007)	0
**Non-JSY institutional deliveries**	-0.500	(-0.768, -0.254)	0
**JSY-supported institutional deliveries**	-0.119	(-0.288, 0.128)	0.298

With regard to the association between MMR and JSY total expenses, the following model was considered.

$$\log(MMR_{d,t}) = \beta_0 + \beta_1 Urban_d + \beta_2 Lit_d + \beta_3 ANC3_{d,t} + \beta_4 NJSY_{d,t} + \beta_5 Exp_{d,t} + \alpha_t + \eta_d$$

The district-specific slope on $Exp_{d,t}$ is omitted. The coefficient estimates are presented in Webtable **7**. Similar to previous finding, no significant relationship was found between MMR and JSY total expenses.

Webtable 7 Estimated fixed effect coefficients from the multilevel regression model without district-specific random slopes.

	Estimated Coefficients	CI	P-values
**Intercept**	6.634	(6.466, 6.798)	0
**Literacy**	-0.002	(-0.005, 0.001)	0.172
**Urban**	-0.0135	(-0.017, -0.010)	0
**ANC3**	-0.009	(-0.011, -0.007)	0
**Non-JSY institutional deliveries**	-0.495	(-0.766, -0.250)	0.002
**JSY total annual expenses**	0.000	(-7.233e-07, 3.215e-06)	0.386

References

1. Held L, Schrödle B, Rue H: **Posterior and Cross-validatory Predictive Checks: A Comparison of MCMC and INLA**. In: *Statistical Modelling and Regression Structures.* edn. Edited by Kneib T, Tutz G: Physica-Verlag HD; 2010: 91-110.
